# Moderate DNA hypomethylation suppresses intestinal tumorigenesis by promoting caspase-3 expression and apoptosis

**DOI:** 10.1038/s41389-021-00328-9

**Published:** 2021-05-04

**Authors:** Xiaoya Duan, Yuanyong Huang, Xiaoxing Chen, Wencai Wang, Jiwei Chen, Jialun Li, Wei Yang, Jiwen Li, Qihan Wu, Jiemin Wong

**Affiliations:** 1 Shanghai Key Laboratory of Regulatory Biology, Fengxian District Central Hospital-ECNU Joint Center of Translational Medicine, Institute of Biomedical Sciences and School of Life Sciences, East China Normal University, Shanghai, 200241 China; 2 Key Laboratory of Reproduction Regulation of NPFPC, SIPPR, IRD, Fudan University, Shanghai, 200032 China; 3 Joint Center for Translational Medicine, Fengxian District Central Hospital, 6600th Nanfeng Road, Fengxian District, Shanghai, 201499 China

**Keywords:** Epigenetics, Epigenetics

## Abstract

Global DNA hypomethylation is a most common epigenetic alteration in human neoplasia. However, accumulative evidence shows that global DNA hypomethylation impacts tumorigenesis in a tissue-specific manner, promoting tumorigenesis in some but suppressing tumorigenesis in others including colorectal cancer. The underlying mechanisms, especially how DNA hypomethylation suppresses tumorigenesis, remain largely unknown. Here, we investigate how DNA hypomethylation affects intestinal tumorigenesis by using an *Uhrf1* tandem tudor domain knockin mutant mouse model (*Uhrf1*^*ki/ki*^) that exhibits a moderate ~10% reduction of global DNA methylation. We found that both chemical-induced colorectal carcinogenesis and *Apc* loss of heterozygosity (LOH)-induced intestinal tumorigenesis are substantially suppressed in the *Uhrf1* mutant mice. Furthermore, unlike *Dnmt1* hypomorphic mice in which DNA hypomethylation suppresses the incidence of macroscopic intestinal tumors but promotes the formation of microadenoma in *Apc*^*Min*/+^ background, *Uhrf1*^*ki/ki*^*/Apc*^*Min/*+^ mice have markedly reduced incidence of both microadenoma and macroadenoma. DNA hypomethylation does not appear to affect *Apc* LOH, activation of the Wnt or Hippo pathway, or tumor cell proliferation, but acts cooperatively with activated Wnt pathway to enhance the caspase-3 gene expression, activation, and apoptosis. Furthermore, increased caspase-3 expression correlates with DNA hypomethylation within the caspase-3 enhancer regions. Taken together, we present a new mouse model for investigating the role of and the molecular mechanisms by which DNA hypomethylation suppresses intestinal tumorigenesis. Our finding that a moderate DNA hypomethylation is sufficient to suppress intestinal tumorigenesis by promoting caspase-3 expression and apoptosis sheds new light on DNA-methylation inhibitor-based colorectal cancer therapeutics.

## Introduction

Genome-wide DNA hypomethylation and concomitant locus-specific DNA hypermethylation are among the most common molecular alterations in human neoplasia^1–5^. These DNA-methylation alterations often occur in the early stage of cancer and is believed to promote cancer pathogenesis through tumor-suppressor gene silencing via promoter hypermethylation and/or through proto-oncogene activation and increased chromosomal instability as a result of global hypomethylation^5–10^. A causal role for global DNA hypomethylation in tumorigenesis was demonstrated by using *Dnmt1* hypomorphic mouse models, which shows genome-wide DNA hypomethylation in all tissues due to a substantially reduced *Dnmt1* expression^11^. These mutant mice developed aggressive T-cell lymphomas at age 4–8 months^11,12^. However, accumulative evidence with various *Dnmt1* hypomorphic mouse models indicate that, while DNA hypomethylation promotes T- and B-cell lymphomas^11,12^, brain tumors^13^, the early stage of prostate cancer^14^, micro-adenomas of intestine and live tumors^15^, it also strongly suppresses overall tumorigenesis in prostate cancer^14^, pancreatic acinar cell tumors^16^, and the macroadenoma of intestine tumors^12,15,17–19^. In fact, weekly treatment with low doses of DNA-methylation inhibitor 5-azaC was shown to almost completely suppress the intestinal adenomas in *Apc*^Min/+^ mice harboring only one wild-type *Dnmt1* allele^20^, providing the first evidence that DNA hypomethylation can actually suppress tumorigenesis.

How global DNA hypomethylation promotes tumorigenesis in some but suppresses in other tissues has become a subject of intensive studies. Because global DNA hypomethylation has been linked to an increased mutation rate^21^, increased genome instability^22^, impaired epigenetic silencing^3,8,23,24^, and activation of interferon pathways^25,26^, it has been proposed that global DNA hypomethylation promotes tumorigenesis in cells that rely on genome instability and suppresses tumorigenesis in cells that are dependent on epigenetic silencing of tumor-suppressor genes. However, exactly how global DNA hypomethylation suppresses intestinal tumorigenesis remains largely unknown. Furthermore, because all global DNA hypomethylation studies reported so far rely on the use of *Dnmt1* hypomorphic mice with substantially reduced levels of DNMT1 proteins, a DNMT1-dependent but DNA-methylation-independent function in tumorigenesis could not be completely ruled out. Thus, a new *Dnmt1*-independent DNA hypomethylation model is needed and can be valuable for testing if the observed tissue-specific effect on tumorigenesis is indeed attributed to global DNA hypomethylation and/or reduced DNMT1 proteins.

Ubiquitin-like with PHD and RING finger domains 1 (UHRF1) has been recognized as a key accessory factor essential for DNMT1-mediated DNA maintenance methylation^27,28^. As a multi-structural and functional domain protein, UHRF1 contains at least five distinct domains from the N- to C-terminus: ubiquitin-like domain, tandem tudor domain (TTD), plant homeo-domain (PHD), SET and RING-associated domain, and RING domain. These domains act cooperatively in binding hemi-methylated CpG sites in newly replicated DNA and histones in targeting DNMT1 to DNA replication foci to carry out DNA maintenance methylation^27,29–34^. The TTD domain shows high affinity of binding histone H3 with di- or tri-methylated lysine 9 (H3K9me2/3)^32,35–37^ and methylated DNA ligase 1^38^. We previously reported the generation of a mutant mouse strain that carried mutations changing tyrosine 187 and proline 188 in TTD to alanine^39^. These mutations abolished UHRF1 binding to H3K9me2/3 and DNA ligase 1. The resulting mutant mice that we named *Uhrf1-TTD-KI* or *Uhrf1*^*ki/ki*^ hereafter are viable and grossly normal. However, the mutant mice were found to exhibit an ~10% reduction of global DNA methylation in various tissues^39^. Thus, the *Uhrf1-TTD-KI* mice may serve as a new model for investigating the role of DNA hypomethylation in tumorigenesis.

In this study, we used *Uhrf1-TTD-KI* mice to investigate and demonstrate that DNA hypomethylation substantially suppresses intestinal tumorigenesis induced by chemical carcinogens or genetic mutation of the *Apc* gene in *Apc*^min/+^ mice. While previous studies with *Dnmt1* hypomorphic mice conclude that DNA hypomethylation suppresses macroadenoma but promotes microadenoma in *Apc*^min/+^ background, we find that both intestinal macroadenoma and microadenoma are markedly suppressed in *Uhrf1*^*ki/ki*^*/Apc*^*Min/*+^ mice. Importantly, we present evidence that DNA hypomethylation in *Uhrf1-TTD-KI* suppresses intestinal tumorigenesis by inducing caspase-3 expression and apoptosis.

## Results

### *Uhrf1*-TTD-KI mice have normal intestinal tissues despite of global DNA hypomethylation

**Fig. 1:**
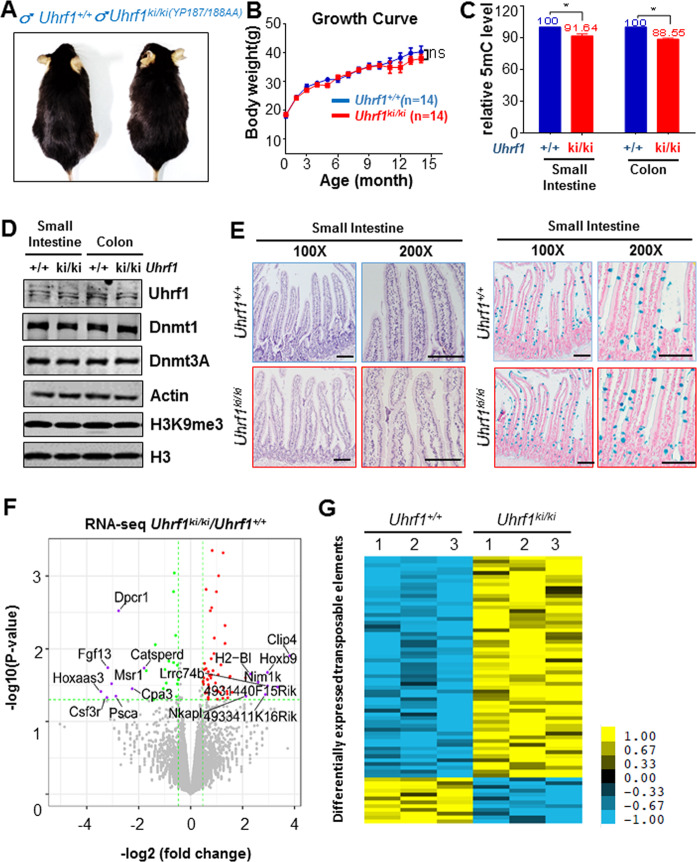
The *Uhrf1*^*ki/ki*^ mice are grossly normal with a moderate DNA hypomethylation in intestinal tissues. **A** Representative images of *Uhrf1*^+/+^ and *Uhrf1*^*ki/ki*^ mice. **B** Growth curves of *Uhrf1*^+/+^ and *Uhrf1*^*ki/ki*^ mice. Data were based on 14 pairs of littermates of *Uhrf1*^+/+^ and *Uhrf1*^*ki/ki*^ mice. ns no significant. Error bars, S.E. **C** Relative levels of DNA methylation in small intestine and colon tissues from *Uhrf1*^+/+^ and *Uhrf1*^*ki/ki*^ mice. Genomic DNA was prepared from the epithelium of small intestines and colons of three pairs of *Uhrf1*^*ki/ki*^ and *Uhrf1*^+/+^ littermate mice and subjected to measurement of 5mC by HPLC. *Uhrf1*^+/+^, *n* = 3; *Uhrf1*^*ki/ki*^, *n* = 3. Error bars, S.E. *p < 0.05. **D** Western blot analysis showing the levels of Uhrf1, Dnmt3a, and Dnmt1 proteins and H3K9me3 in small intestine and colon tissues from *Uhrf1*^*ki/ki*^ and *Uhrf1*^+/+^ mice. The protein extracts were prepared from the epithelium cells derived from small intestines and colons of 8-week-old mice. Actin and H3 served as a loading control. **E** H&E staining (left) and Alcian blue staining (right) of the small intestine tissues from *Uhrf1*^+/+^ and *Uhrf1*^*ki/ki*^ mice. Shown are samples from 8-week mice. Scale bar, 100 μm. **F** Volcano plot of RNA-seq data. RNA-seq analysis was performed with RNAs from intestinal epithelium cells derived from three pairs of *Uhrf1*^+/+^ and *Uhrf1*^*ki/ki*^ mice. Sixty-five upregulated genes (red) and 33 downregulated genes (green) were identified in small intestinal epithelium from *Uhrf1*^*ki/ki*^ mice when compared with *Uhrf1*^+/+^. **G** Heat maps showing differentially expressed transposable elements in small intestinal epithelium from *Uhrf1*^+/+^ and *Uhrf1*^*ki/ki*^ mice.

We previously reported the *Uhrf1-TTD-KI* mouse model with both 187 tyrosine and 188 proline residues in TTD mutated to alanine^39^. The homozygous mutant mice are viable and grossly normal, despite an ~10% reduction of global DNA methylation in liver, brain, and heart tissues. To examine if this mouse model is suitable for investigating how DNA hypomethylation affects intestinal tumorigenesis, we first obtained wild-type, heterozygous and homozygous mutant mice by crossing between heterozygote mice. The genotypes of the resulting mice were determined by both DNA sequencing and PCR-based genotyping (Supplementary Fig. 1A, B). Through observing a large number of resulting littermate mice, we found no significant difference between the wild-type, heterozygous (data not shown), and homozygous *Uhrf1*-*TTD-KI* mice in gross morphology and the growth rate within 14 months of observation (Fig. 1A, B). At the end of experiments, all mice were examined and found to be free of intestinal tumors and tumors in other tissues. However, measurement of DNA-methylation (5mC) levels from three pairs of littermates by quantitative high-pressure liquid chromatography (HPLC) analysis revealed an average of 8.4% reduction of 5mC in intestines and 11.5% reduction of 5mC in colons of mutant mice compared to the corresponding wild-type littermates (Fig. 1C), a result consistent with our previous measurement of 5mC levels in other tissues^39^. The same analysis revealed no difference in DNA methylation in both small intestine and colon tissues between wild-type and heterozygote *Uhrf1*-*TTD-KI* mice (data not shown). The defect in DNA methylation could be attributed to impaired binding of H3K9me2/3 and/or methylated DNA ligase 1 by the mutated Uhrf1 protein, which consequently led to reduced Dnmt1 DNA maintenance activity, because Western blot analysis detected no difference between wild-type and mutant mice in the protein levels of Uhrf1, Dnmt1 and Dnmt3a, and H3K9me3 in the small intestine and colorectal tissues (Fig. 1D). Furthermore, hematoxylin and eosin (H&E) staining of tissue sections showed that *Uhrf1-TTD-KI* mice had normal intestinal crypts and villi as observed in the wild-type mice (Fig. 1E, left panel). BrdU incorporation assay showed normal proliferation of intestinal stem and transient cells of mutant mice (Supplementary Fig. 1C, left panel), as indicated by equal BrdU labeling of the intestinal crypt cells of both wild-type and *Uhrf1-TTD-KI* mice 5 h after BrdU injection. Furthermore, at 50 h post BrdU injection, the BrdU-labeled cells were found to account for ~1/2 of the villi in both the wild-type and the *Uhrf1-TTD-KI* mice, indicating normal proliferation and migration of the intestinal epithelial cells in *Uhrf1*-*TTD-KI* mice (Supplementary Fig. 1C, right panel). Consistent with the BrdU labeling result, quantitative real-time PCR (qRT-PCR) analysis revealed a similar level of stem-cell-specific transcripts of *Lgr5* in both *Uhrf1*-*TTD-KI* and wild-type mice (Supplementary Fig. 1D). Moreover, the alcian blue staining showed that the intestinal epithelium of *Uhrf1*-*TTD-KI* mice was able to differentiate into mature goblet cells as effective as the wild-type mice (Fig. 1E, right panel and Supplementary Fig. 1E). qRT-PCR analysis also revealed a similar level of *Lyz1* mRNA, a marker of mature Paneth cells, in wild-type and *Uhrf1-TTD-KI* mice (Supplementary Fig. 1F). Taken together, we conclude that, despite of an ~10% reduction of global DNA methylation, the small intestine and colorectal tissues in *Uhrf1-TTD-KI* mice develop and differentiate normally and are free of spontaneous tumors.

To characterize the potential effect of a moderate DNA hypomethylation on gene expression in the small intestine, epithelial cells were prepared from small intestine tissues of three pairs of wild-type and *Uhrf1*-*TTD-KI* littermate mice. Total RNAs were prepared from epithelial cells and used for gene expression profiling by RNA-seq. This analysis revealed that DNA hypomethylation did not significantly alter the patterns of gene expression, because RNA-seq analysis only identified 60 upregulated genes and 26 downregulated genes in small intestinal epithelial cells from *Uhrf1*-*TTD-KI* compared to those from the wild-type control by using *p* value < 0.05 and fold change >1.5 as cut-off (Fig. 1F and a list of the genes in Supplementary Table 1). Six differentially expressed genes, *Acer1, Ceacam10, Fmr1nb, Msr1, Dpcr1*, and *Slc4a11*, were picked for validation by qRT-PCR and the results were in general consistent with the RNA-seq data (Supplementary Fig. 1G). We also compared the expression profile of transposon sequences, because DNA methylation is known to play a role in genome stability by suppressing various transposon elements in the mouse genome. Again, only 70 transposable elements were found to have a moderately altered expression (Fig. 1G and Supplementary Table 2). We therefore conclude that the moderate DNA hypomethylation in *Uhrf1*-*TTD-KI* has a relatively mild effect on gene expression in intestinal tissues. Furthermore, since only a small percentage of transposon elements was affected, this moderate DNA hypomethylation does not appear to significantly impact genome stability.

### Moderate DNA hypomethylation in *Uhrf1-TTD-KI* mice is sufficient to suppress chemically induced colorectal cancer

**Fig. 2:**
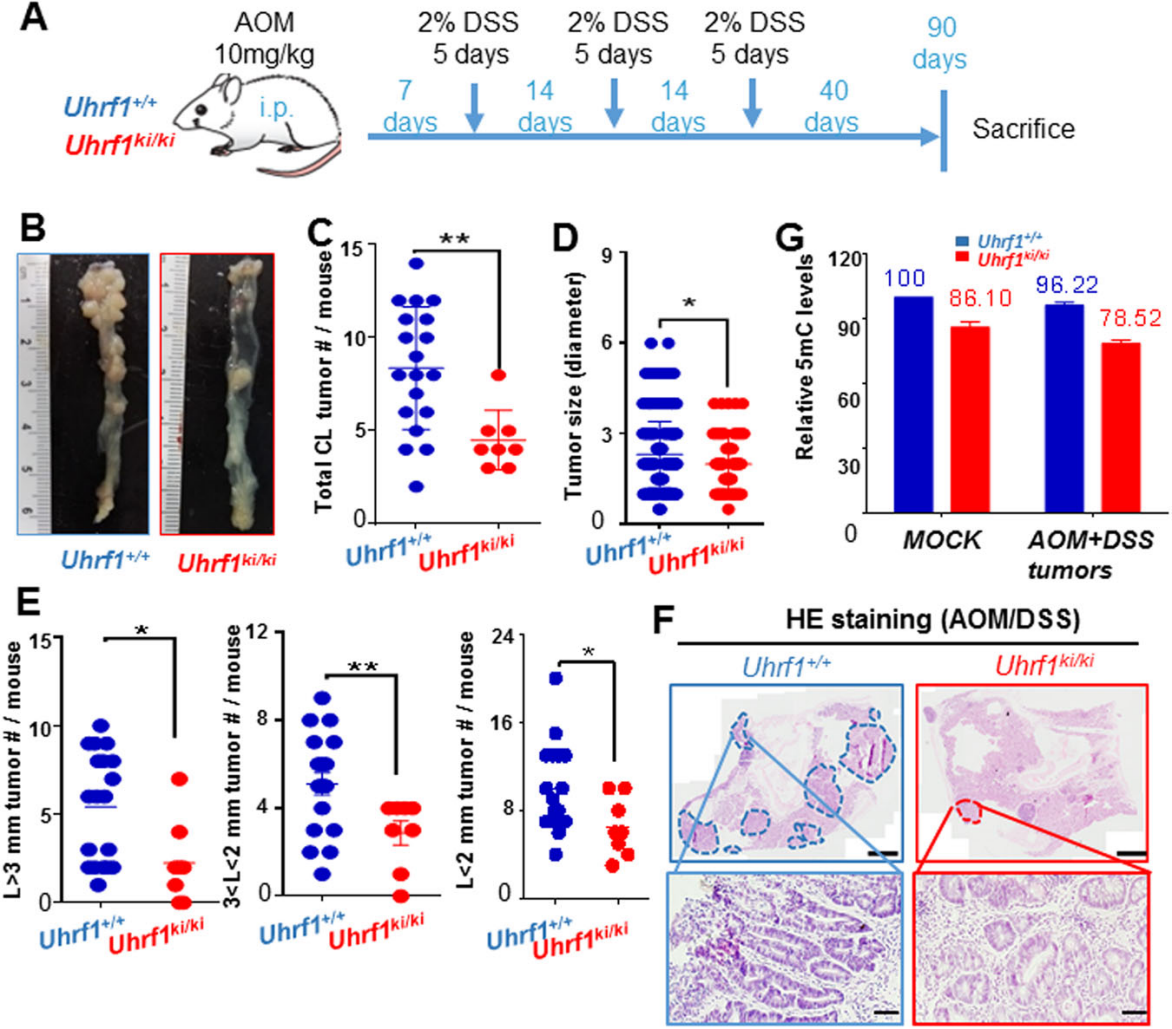
*Uhrf1*^*ki/ki*^ mice are resistant to AOM/DSS-induced colorectal tumorigenesis. **A** Experimental scheme for induction of colorectal tumors in *Uhrf1*^+/+^ and *Uhrf1*^*ki/ki*^ mice by AOM/DSS treatment. **B** Representative macroscopic images of AOM/DSS-induced colorectal tumors from *Uhrf1*^+/+^ and *Uhrf1*^*ki/ki*^ mice. Comparison of total number of tumors per mouse (**C**) and sizes of tumors (**D**) from AOM/DSS-treated *Uhrf1*^+/+^ and *Uhrf1*^*ki/ki*^ mice. The tumor size was measured by stereomicroscope. Error bars, S.E. *Uhrf1*^+/+^, *n* = 19; *Uhrf1*^*ki/ki*^, *n* = 8. **p < 0.01; *p < 0.05. **E** Tumors were classified according to the size ranges (*L* > 3 mm; 3 > *L* > 2 mm, and *L* < 2 mm) and plotted as number of tumors per mouse. *L*: diameter of tumor. Error bars, S.E. *Uhrf1*^+/+^ (*n* = 19); *Uhrf1*^*ki/ki*^ (*n* = 8). **p < 0.01; *p < 0.05. **F** Representative H&E staining images of colon cross-sections. Tumor lesions in *Uhrf1*^+/+^ mice and *Uhrf1*^*ki/ki*^ mice are marked in blue or red circle, respectively. Upper scale bars, 2 mm. Bottom scale bars, 50 μm. **G** DNA-methylation levels in colon tissues from control *Uhrf1*^+/+^ and *Uhrf1*^*ki/ki*^ mice or tumors from AOM/DSS-treated *Uhrf1*^+/+^ and *Uhrf1*^*ki/ki*^ mice. For control group, *Uhrf1*^+/+^, *n* = 3; *Uhrf1*^*ki/ki*^, *n* = 3. For AOM/DSS-treated mice, *Uhrf1*^+/+^, *n* = 6; *Uhrf1*^*ki/ki*^, *n* = 6. Genomic DNA was prepared from normal epithelium cells or microdissected tumor tissues, and 5mC level was measured by HPLC. Error bars, S.E.

We next tested whether moderate DNA hypomethylation in *Uhrf1*-*TTD-KI* mice is sufficient to impact colorectal tumorigenesis induced by a combined treatment of azoxymethane (AOM) and dextran sulfate sodium (DSS). AOM, the metabolite of 1,2-dimethylhydrazine, is a specific colon carcinogen, whereas DSS is a chemical that induces colitis in rodents. The combined use of AOM and DSS can result in 100% incidence of colorectal tumors in rodents^40,41^. We therefore employed a published protocol as illustrated in Fig. 2A to induce colorectal tumors by AOM/DSS in wild-type and *Uhrf1*-*TTD-KI* mice^42^. At the end of 90 days of experiments, the mice were sacrificed and colorectal tissues were dissected for analysis of tumor numbers and sizes. We found that *Uhrf1*-*TTD-KI* mice had a much lower tumor burden than wild-type mice (Fig. 2B, C). Moreover, the *Uhrf1*-*TTD-KI* mice had a significantly reduced number of large-size tumors (tumors with diameter *L* ≥ 3 mm and 3 > *L* ≥ 2 mm) than wild-type mice (Fig. 2D–E), although the difference in tumor lesions with a size smaller than 2 mm is less significant. This observation was further confirmed by H&E staining, which revealed less tumor lesions in colorectal tissues from *Uhrf1*-*TTD-KI* mice than those from the wild-type mice (Fig. 2F). In addition, H&E staining also confirmed that tumors in *Uhrf1*-*TTD-KI* mice were smaller in size than those in the wild-type mice (Fig. 2F). Thus, moderate DNA hypomethylation can substantially suppress chemically induced colorectal tumorigenesis in mice.

As tumorigenesis often results in global DNA hypomethylation, we assessed if the pattern of differential DNA methylation between *Uhrf1*-*TTD-KI* and wild-type mice was persisted in tumors. Genomic DNA was prepared from AOM/DSS-untreated colorectal tissues and AOM/DSS-induced colorectal tumors that were microdissected from both wild-type and *Uhrf1*-*TTD-KI* mice, and the levels of 5mC were quantitatively measured by HPLC. The results in Fig. 2G showed that there was a reduced level of DNA methylation in tumors vs. normal colorectal tissues. Furthermore, the level of DNA methylation in tumors from *Uhrf1*-*TTD-KI* mice was substantially lower than that in tumors from the wild-type mice, indicating that reduced DNA methylation in *Uhrf1-TTD-KI* mice was inherited to tumor cells.

Taken together, this chemically induced colorectal tumor model demonstrates that moderate DNA hypomethylation is sufficient to substantially suppress colorectal tumorigenesis and tumor progression in mice.

### Moderate DNA hypomethylation also substantially suppresses tumorigenesis and tumor progression in *Apc*^Min/+^ mice

**Fig. 3:**
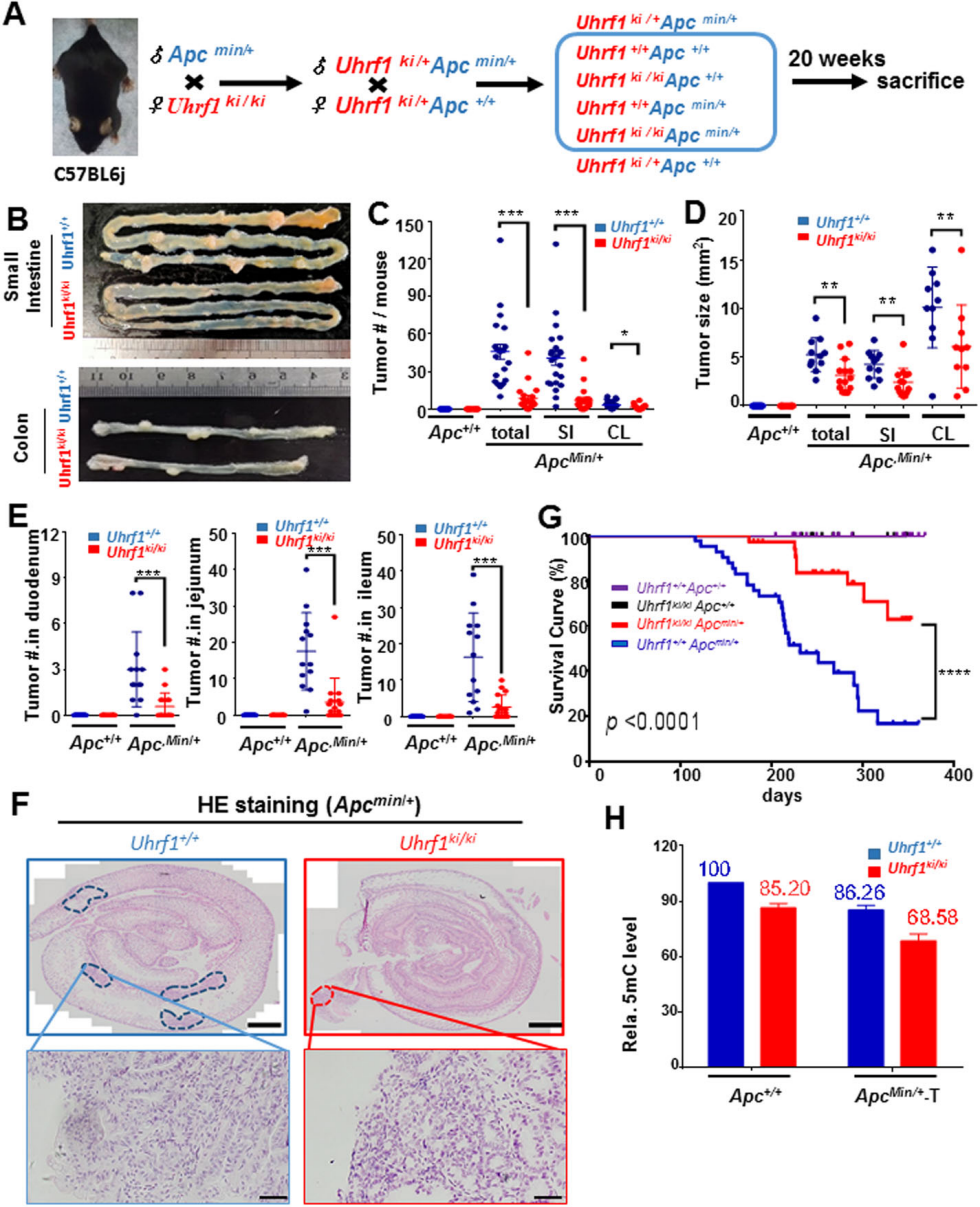
*Uhrf1*^*ki/ki*^ mutation markedly suppresses intestinal tumorigenesis in *Apc*^Min/+^mice. **A** Mating strategy for generation of *Uhrf* ^*ki/ki*^ mutation in *Apc*^*min*/+^ mice. **B** Representative macroscopic images of small intestinal tumors and colorectal tumors from *Uhrf1*^+/+^*/Apc*^*min*/+^ and *Uhrf1* ^*ki/ki*^*/Apc*^*min*/+^ mice. The number of intestinal tumors per mouse (**C**) and the sizes of intestinal tumors (**D**). *Uhrf1*^+/+^*/Apc*^+/+^, *n* = 21; *Uhrf1* ^*ki/ki*^*/Apc*^+/+^, *n* = 16; *Uhrf1*^+/+^*/Apc*^*min*/+^, *n* = 23; and *Uhrf1* ^*ki/ki*^*/Apc*^*min/*+^, *n* = 20 mice. Tumors were classified as total number of tumor, numbers of tumors in small intestine (SI) or colon (CL). Error bars, S.E. ***p < 0.001; **p < 0.01; *p < 0.05. **E** The numbers of tumors per mouse in duodenum, jejunum, and ileum. *Uhrf1*^+/+^*/Apc*^+/+^, *n* = 21; *Uhrf1*^*ki/ki*^*/Apc*^+/+^, *n* = 16; *Uhrf1*^+/+^*/Apc*^*min*/+^, *n* = 23; and *Uhrf1*^*ki/ki*^*/Apc*^*min*/+^, *n* = 20 mice. Error bars, S.E. ***p < 0.001. **F** Representative H&E staining images of small intestines. Tumor lesions in *Uhrf1*^+/+^*/Apc*^*min*/+^ and *Uhrf1*^*ki/ki*^*/Apc*^*min/*+^ mice are shown in blue circle and red circle, respectively. Upper scale bars, 2 mm. Bottom scale bars, 50 μm. **G** Kaplan–Meier curves for overall survival. *Uhrf1*^+/+^*/Apc*^+/+^, *n* = 27; *Uhrf1*^*ki/ki*^*/Apc*^+/+^, *n* = 22; *Uhrf1*^+/+^/*Apc*^*min*/+^, *n* = 37; *Uhrf1*^*ki/ki*^*/Apc*^*min*/+^, *n* = 35 mice. Error bars, S.E. ****p < 0.0001. **H** DNA-methylation levels in normal intestinal tissues or tumors. *Uhrf1*^+/+^*/Apc*^+/+^, *n* = 3; *Uhrf1* ^*ki/ki*^*/Apc*^+/+^, *n* = 3; *Uhrf1*^+/+^*/Apc*^*min*/+^, *n* = 6; and *Uhrf1*^*ki/ki*^*/Apc*^*min*/+^, *n* = 3 mice. Genomic DNA was prepared from the small intestinal epithelium cells, and 5mC level was measured by HPLC. Error bars, S.E.

Germline mutation of the adenomatous polyposis coli (APC) gene causes familial adenomatous polyposis, a hereditary tumorous predisposition syndrome, which is diagnosed by detection of adenomatous polyps^43,44^. The multiple intestinal neoplasia (Min) mice (*Apc*^min/+^) carry a T to A transversion mutation in the *Apc* gene that results in a premature stop codon at codon 850^45,46^. Like humans with germline mutations in APC, *Apc*^min/+^ mice are predisposed to intestinal adenoma formation and have been extensively used as a model for intestinal tumorigenesis. To investigate how *Uhrf1*-*TTD-KI*-induced DNA hypomethylation affects intestinal tumorigenesis in *Apc*^min/+^ mice, male *Apc*^min/+^ mice were crossed with female *Uhrf1*-*TTD-KI* mice according to a scheme illustrated in Fig. 3A to generate six different genotypes of mice. At the age of 20 weeks, mice were sacrificed and the number and size of intestinal tumors were analyzed. As expected, no intestinal and colon tumors were observed in *Apc*^+/+^ mice for *Uhrf1*^+/+^, *Uhrf1*^+*/ki*^ and *Uhrf1*^*ki/ki*^ genetic background (data not shown). Importantly, comparison of *Uhrf1*^+/+^/*Apc*^*Min/*+^ and *Uhrf1*^*ki/ki*^*/Apc*^*Min/*+^ mice revealed a substantially reduced tumor burden in *Uhrf1*^*ki/ki*^*/Apc*^*Min/*+^ mice (Fig. 3B, C). The average number of small intestinal and colorectal tumors in *Uhrf1*^+/+^*/Apc*^*Min/*+^ is 44.04 and this is reduced to 8.6 in *Uhrf1*^*ki/ki*^*/Apc*^*Min/*+^ mice (Fig. 3C). Tumor reduction occurred primarily in small intestines, whereas much less tumors were observed in colons (Fig. 3C). Furthermore, the sizes of tumors in *Uhrf1*^*ki/ki*^*/Apc*^*Min/*+^ mice were also much smaller in comparison to tumors in *Uhrf1*^+/+^*/Apc*^*Min/*+^ (Fig. 3D and Supplementary Fig. 2). We also analyzed the tumor status according to the anatomical structure (duodenum, jejunum, and ileum). This analysis revealed a more dramatic reduction of tumor numbers in the ileum and jejunum than duodenum (Fig. 3E). To more precisely define the number and sizes of intestinal tumors, we also carried out H&E staining of tissue sections. Representative results in Fig. 3F confirmed a substantial reduction of both tumor number and tumor size in the small intestines of *Uhrf1*^*ki/ki*^*/Apc*^*Min*/+^ mice. Consistent with a substantial reduction in tumor burden, the *Uhrf1*^*ki/ki*^/*Apc*^*Min*/+^ mice had a significantly prolonged lifespan than *Uhrf1*^+/+^/*Apc*^*Min*/+^ mice (Fig. 3G). Similar to the results from AOM/DSS-induced tumor model, measurement of 5mC levels in normal tissues from *Apc*^+/+^ background and tumors from *Apc*^*min*/+^ background revealed a substantial reduction of DNA methylation in tumors, and the DNA hypomethylation phenotype is more drastic in tumors from the *Uhrf1*^*ki/ki*^/*Apc*^*Min*/+^ mice (Fig. 3H).

Taken altogether, these results demonstrate that DNA hypomethylation caused by *Uhrf1-TTD-KI* mutation can markedly suppress intestinal tumorigenesis in *Apc*^*Min/+*^ mice.

### DNA hypomethylation caused by *Uhrf1-TTD-KI* mutation does not affect *Apc* loss of heterozygosity and activation of Wnt and Hippo pathways

Tumorigenesis in small intestine and colon tissues in *Apc*^*Min*/+^ mice is known to associate with loss of heterozygosity (LOH) of wild-type *Apc* allele^47,48^, which results in activation of both Wnt and Hippo pathways^49–51^. Having established that DNA hypomethylation caused by *Uhrf1-TTD-KI* mutation substantially suppresses colorectal tumorigenesis induced by AOM/DSS and intestinal tumorigenesis in *Apc*^*Min*/+^ mice, we next investigated the potential mechanisms. To this end, we first analyzed if DNA hypomethylation affected LOH of *Apc*. According to previous publications, wild-type *Apc* allele and *Apc*^*Min*^ allele could be distinguished by PCR amplification followed by restriction enzyme digestion by HindIII, which generated a 144-bp DNA fragment for the *Apc*^*Min*^ allele and a 123-bp fragment for the wild-type *Apc* allele (Supplementary Fig. 3A). Using this assay, we detected LOH of the *Apc* allele in small intestine tumors from both *Uhrf1*^+/+^/*Apc*^*Min*/+^ and *Uhrf1*^*ki/ki*^/*Apc*^*Min*/+^ mice (Supplementary Fig. 3B). Consistent with the observed LOH of *Apc* allele, Western blot analysis revealed elevated levels of β-catenin protein in tumors compared to the normal tissues in both *Uhrf1*^+/+^*/Apc*^*Min*/+^ and *Uhrf1*^*ki/ki*^*/Apc*^*Min*/+^ mice (Supplementary Fig. 3C). Similarly, Western blot analysis revealed elevated levels of YAP protein in tumors compared to the normal tissues in both *Uhrf1*^+/+^*/Apc*^*Min*/+^ and *Uhrf1*^*ki/ki*^*/Apc*^*Min*/+^ mice, in agreement with previous observation^49^ (Supplementary Fig. 3D). Furthermore, while immunohistochemistry (IHC) analysis revealed cytoplasmic localization of β-catenin in normal tissues, IHC detected obvious nuclear localization of β-catenin in tumor tissues from both *Uhrf1*^+/+^*/Apc*^*Min*/+^ and *Uhrf1*^*ki/ki*^*/Apc*^*Min*/+^ mice (Supplementary Fig. 4A, B). Together, these data suggest that there is no significant difference in LOH of *Apc* and activation of Wnt and Hippo pathways in both *Uhrf1*^+/+^*/Apc*^*Min*/+^ and *Uhrf1*^*ki/ki*^*/Apc*^*Min*/+^ mice.

### DNA hypomethylation caused by *Uhrf1-TTD-KI* mutation does not affect intestinal tumor stem cells and tumor cell proliferation

To analyze if DNA hypomethylation suppresses intestinal tumorigenesis in *Uhrf1*^*ki/ki*^*/Apc*^*Min*/+^ mice by affecting tumor stem cells, we examined the levels of intestinal stem-cell marker genes *Lgr5*, *Ascl2*, and *Sox9* by qRT-PCR. The results showed that compared with the normal tissues, these genes were highly expressed in the tumor tissues, but there was no significant difference between tumors from *Uhrf1*^+/+^*/Apc*^*Min*/+^ and *Uhrf1*^*ki/ki*^*/Apc*^*Min*/+^ mice (Supplementary Fig. 5A).

We also analyzed if potential difference in cell proliferation contributes to suppressed tumorigenesis in *Uhrf1*^*ki/ki*^*/Apc*^*Min*/+^ mice. However, IHC analysis revealed no difference in the level of PCNA protein between *Uhrf1*^+/+^*/Apc*^*Min*/+^ and *Uhrf1*^*ki/ki*^*/Apc*^*Min*/+^ mice, even though the levels of PCNA were increased in tumors from both mice (Supplementary Fig. 5B, C). This observation was further confirmed by Western blot analysis in Supplementary Fig. 5D, showing that although PCNA was highly expressed in tumors, its levels were similar between *Uhrf1*^+/+^*/Apc*^*Min*/+^ and *Uhrf1*^*ki/ki*^*/Apc*^*Min*/+^ mice regardless of normal or tumor tissues. Similarly, IHC analysis showed no significant difference of Ki67, another marker for cell proliferation, between *Uhrf1*^+/+^*/Apc*^*Min*/+^ and *Uhrf1*^*ki/ki*^*/Apc*^*Min*/+^ mice in both normal and tumor tissues (Supplementary Fig. 5E, F).

### DNA hypomethylation caused by *Uhrf1-TTD-KI* mutation is not sufficient to activate interferon pathway

Recent studies provide evidence that DNMT inhibitor 5-Aza-C exerts an antitumor therapeutic effect by inducing activation of the interferon pathway^25,26^. This is mediated by DNA hypomethylation-induced expression of endogenous retrovirus and formation of double-stranded RNAs, which activates the interferon signaling pathway through toll-like receptor signaling and induces apoptosis of tumor cells. To test if DNA hypomethylation in *Uhrf1*^*ki/ki*^*/Apc*^*Min*/+^ mice activated the interferon pathway, we measured the transcript levels of the key members of toll-like receptor signaling and interferon pathways by qRT-PCR. Results in Supplementary Fig. 6 showed no obvious difference in expression of toll-like receptor signaling and interferon pathway components *Irf3/7/9*, *Stat1*, and *Isg15* between *Uhrf1*^+/+^*/Apc*^*Min*/+^ and *Uhrf1*^*ki/ki*^*/Apc*^*Min*/+^ mice in either normal or tumor tissues. Thus, the moderate DNA hypomethylation observed in *Uhrf1*-*TTD-KI* mice is not sufficient to activate the interferon pathway in *Apc*^*Min*/+^ background. This observation is consistent with limited alteration of transposable element expression in *Uhrf1*-*TTD-KI* mice (Fig. 1F). Thus, we conclude that suppression of intestinal tumorigenesis in *Apc*^*Min*/+^ mice by *Uhrf1-TTD-KI*-induced DNA hypomethylation is unlikely due to activation of the antiviral interferon pathway.

### DNA hypomethylation caused by *Uhrf1*-*TTD-KI* mutation promotes intestinal epithelium apoptosis in *Apc*^*Min*/+^ mice

**Fig. 4:**
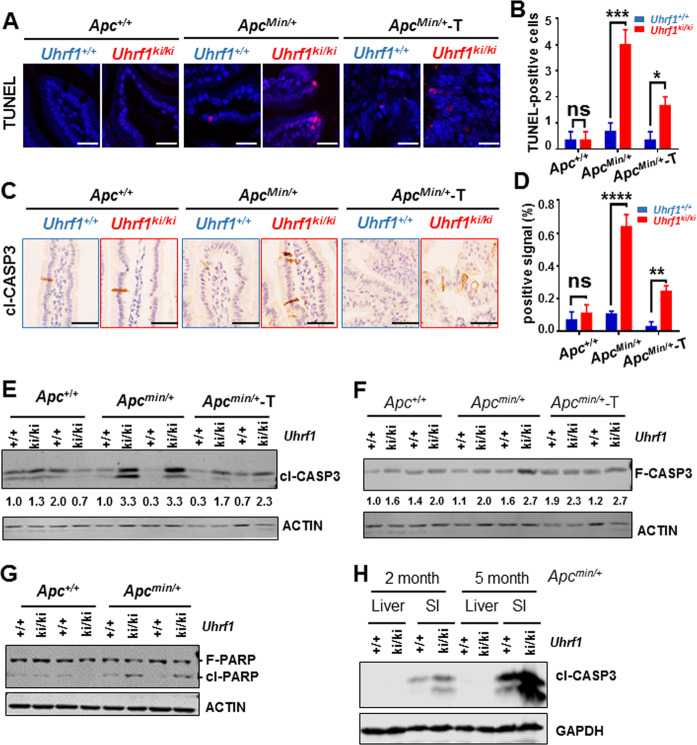
Elevated apoptosis and active caspase-3 in the intestinal tissues of *Uhrf1*^*ki/ki*^*/Apc*^*min*/+^ mice. **A, B** TUNEL assay showing levels of apoptosis in the intestinal sections from different mice. Representative confocal microscope images of TUNEL assay (**A**). Nuclei were stained with DAPI. Scale bar, 25 μm. Quantitative results of TUNEL-positive cells per 0.01 mm^2^ (**B**). Three mice were analyzed for each genotypes. *Apc*^*min*/+^-Tumor, tumor samples from *Uhrf1*^+/+^*/Apc*^*min*/+^ and *Uhrf1*^*ki/ki*^*/Apc*^*min*/+^ mice. Error bars, S.E. **C, D** Representative immunohistochemistry images showing the cleaved-caspase-3 (cl-CASP3) levels in small intestinal tissue sections from different mice (**C**). Scale bar, 50 μm. The number of cl-CASP3-positive cells were analyzed by ImageJ software (**D**). Three mice were analyzed for each genotypes. Error bars, S.E. Western blot results showing the levels of cl-CASP3 (**E**) and full-length caspase-3 (F-CASP3) (**F**) in small intestinal epithelium cells from different mice as indicated. The relative levels of cl-CASP3 and F-CASP3 were determined by ImageJ software. **G** Western blot results showing the levels of cleaved PRAP (cl-PARP) and full-length PARP (F-PARP) proteins in small intestinal epithelium cells from different mice as indicated. Note elevated levels of cl-PARP proteins were observed in small intestinal epithelium cells from *Uhrf1*^*ki/ki*^*/Apc*^*min*/+^ mice. **H** Western blot results showing the levels of cleaved-caspase-3 (cl-CASP3) proteins in liver tissues and small intestine epithelium cells from different mice as indicated. Note that cl-CASP3 was not detected in liver tissues from *Uhrf1*^*ki/ki*^*/Apc*^*min*/+^ mice.

Having excluded cell proliferation and activation of the interferon pathway as potential mechanisms for tumor suppression by *Uhrf1-TTD-KI* mutation, we examined if elevated apoptosis^52,53^ occurred in *Uhrf1*^*ki/ki*^*/Apc*^*Min*/+^ mice. We performed TUNEL assay to compare apoptosis in normal intestinal tissues from *Apc*^+/+^ and *Apc*^*Min*/+^ background and tumor tissues from *Apc*^*Min*/+^ background. Notably, we observed a marked elevation of apoptosis in normal tissues from *Uhrf1*^*ki/ki*^*/Apc*^*Min*/+^ mice in comparison to those from *Uhrf1*^+/+^*/Apc*^*Min*/+^ mice (Fig. 4A, B). In addition, there was also a significantly higher level of apoptosis in the tumor tissues from *Uhrf1*^*ki/ki*^*/Apc*^*Min*/+^ vs. *Uhrf1*^+/+^*/Apc*^*Min*/+^ mice (Fig. 4A, B). In support of the above observation, IHC analysis revealed a significantly elevated level of cleaved-caspase-3 (cl-CAPS-3, the active form of caspase-3)-positive cells in normal tissues from *Uhrf1*^*ki/ki*^*/Apc*^*Min*/+^ vs. *Uhrf1*^+/+^*/Apc*^*Min*/+^ mice (Fig. 4C). Mirroring the TUNEL assay results, quantitative analysis of cl-CAPS-3 revealed that despite a reduced level of cl-CAPS-3-positive cells in tumors vs. normal tissues, the levels of cl-CAPS-3-positive cells remained significantly higher in tumors from *Uhrf1*^*ki/ki*^*/Apc*^*Min*/+^ than tumors from *Uhrf1*^+/+^*/Apc*^*Min*/+^ mice (Fig. 4D). To ensure a correct detection of cl-CAPS-3, we prepared the normal and tumor tissues from small intestines of all four genetic backgrounds and carried out Western blot analysis for both cl-CAPS-3 and the full-length proteins. The representative results in Fig. 4E confirmed a marked increase of cl-CAPS-3 proteins in normal intestinal tissues from *Uhrf1*^*ki/ki*^*/Apc*^*Min*/+^ vs. *Uhrf1*^+/+^*/Apc*^*Min*/+^ mice. In accord with the IHC results, the levels of cl-CASP-3 in tumor tissues from *Uhrf1*^*ki/ki*^*/Apc*^*Min*/+^ mice were clearly lower than that in normal tissues, suggesting a suppressed apoptosis in tumors. Nevertheless, comparison between tumors from *Uhrf1*^*ki/ki*^*/Apc*^*Min*/+^ and *Uhrf1*^+/+^*/Apc*^*Min*/+^ mice revealed a higher level of cl-CAPS-3 in tumors from *Uhrf1*^*ki/ki*^*/Apc*^*Min*/+^ mice. Notably, Western blot analysis revealed an elevated level of full-length caspase-3 that was detected in normal small intestinal tissues of *Uhrf1*-*TTD-KI* mice in comparison to *Uhrf1*-*WT* mice (Fig. 4F). The increase of full-length caspase-3 was more obvious when compared between *Uhrf1*^*ki/ki*^*/Apc*^*Min*/+^ and *Uhrf1*^+/+^*/Apc*^*Min*/+^ mice (Fig. 4F). Thus, while increased full-length caspase-3 levels can be attributed to *Uhrf1*-*TTD-KI* mutation, cleaved caspase-3 and activation of apoptosis seem to occur primarily in *Uhrf1*^*ki/ki*^*/Apc*^*Min*/+^ mice, suggesting that loss of *Apc* somehow leads to cleavage of caspase-3 and activation of apoptosis.

Poly ADP-ribose polymerase (PARP) is an important substrate of cleaved caspase-3^54^. During activation of apoptosis, the 116-kDa PARP is cleaved into 31 and 85-kDa fragments by activated caspase-3. In further support of elevated apoptosis in small intestines of *Uhrf1*^*ki/ki*^*/Apc*^*Min*/+^ mice, Western blot analysis detected an increased level of cleaved PARP in small intestines from *Uhrf1*^*ki/ki*^*/Apc*^*Min*/+^ mice but not in small intestines from *Uhrf1*^+/+^*/Apc*^*Min*/+^ mice or *Uhrf1*^*ki/ki*^ and *Uhrf1*^+/+^ mice (Fig. 4G).

DNA hypomethylation in *Dnmt1* hypomorphic mice has been shown to impact tumorigenesis in a tissue-specific manner, suppressing tumorigenesis in small intestines and promoting tumorigenesis in livers^15^. Having unraveled an increased apoptosis in small intestines of *Uhrf1*^*ki/ki*^*/Apc*^*Min*/+^ mice, we wondered if increased apoptosis also occurs in liver. However, as shown in Fig. 4H, we found that the elevated cl-CAPS-3 levels were specifically detected in small intestinal tissues and were absent in liver tissues from *Uhrf1*^*ki/ki*^*/Apc*^*Min*/+^ mice.

Altogether, these data provide evidence that increased apoptosis occurs specifically in the intestinal tissues and is likely a mechanism for DNA hypomethylation-induced suppression of intestinal tumors in *Uhrf1*^*ki/ki*^*/Apc*^*Min*/+^ mice.

### Enhancer DNA hypomethylation correlates with increased caspase-3 expression in *Uhrf1*^*ki/ki*^*/Apc*^*Min*/+^ mice

**Fig. 5:**
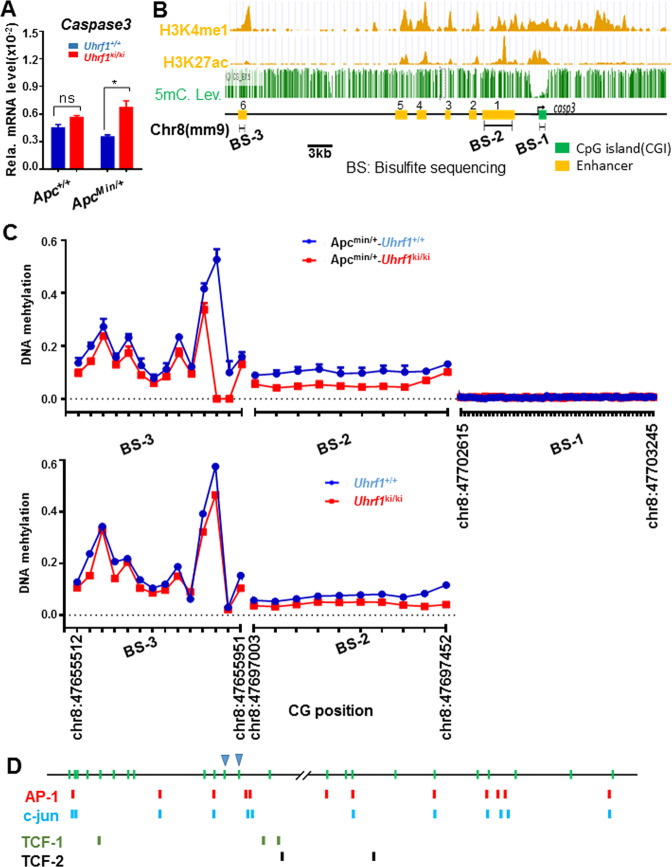
Activation of caspase-3 gene expression in *Uhrf1*^*ki/ki*^*/Apc*^*min*/+^ mice correlates with reduced DNA methylation in the caspase-3 enhancer regions. **A** qRT-PCR analysis showing elevated levels of caspase-3 mRNA in small intestinal epithelium cells from *Uhrf1*^*ki/ki*^*/Apc*^*min/*+^ mice. The levels of caspase-3 mRNA were determined from three pairs of littermate mice and were normalized to *Actin*. *p < 0.05; ns, *p* > 0.05. Three mice were analyzed for each genotypes. Error bars, S.E. **B** Diagrams illustrating the epigenetic marks H3K4me1 and H3K27ac landscapes at the mouse caspase-3 gene. The profiles were derived from small intestinal tissue ChIP-seq data in UCSC genome browser. The putative enhancers, from enhancer 1 to 6, were assigned according to overlapping H3K27ac and H3K4me1 peaks. As a reference, also shown are the DNA-methylation status within or upstream of casepase-3 gene derived from E7.5 mouse embryos (downloaded from Methbank database). **C** Summary of bisulfite sequencing results of DNA-methylation status within promoter, enhancer 1 and enhancer 6 regions. Each CpG sites were sequenced over hundred or thousand times by high-throughput Illumina^®^ (ND102-0102) sequencing and thus the data were highly accurate. Note that the caspase-3 promoter region in small intestinal epithelium cells is essentially unmethylated in both *Uhrf1*^+/+^*/Apc*^*min/*+^ and *Uhrf1*^*ki/ki*^*/Apc*^*min*/+^ mice. **D** Caspase-3 enhancer 1 and enhancer 6 contain multiple binding sites for Wnt-regulated transcription factors TCF1/2, AP-1, and c-jun. The two highly demethylated CpG sites are marked with triangle.

Having observed elevated levels of both cleaved and full-length caspase-3 in the intestinal tissues of *Uhrf1*^*ki/ki*^*/Apc*^*Min*/+^ mice, we next examined if elevated expression of caspase-3 occurs at the level of transcription. Indeed, qRT-PCR analysis revealed an elevated level of caspase-3 mRNA in the small intestinal epithelium of *Uhrf1*^*ki/ki*^*/Apc*^*Min*/+^ mice vs. that of *Uhrf1*^+/+^*/Apc*^*Min*/+^ mice (Fig. 5A). Elevated caspase-3 mRNA was also observed in the small intestinal epithelium of *Uhrf1*^*ki/ki*^ mice vs. that of *Uhrf1*^+/+^ mice, although the results were not statistically significant (Fig. 5A).

We hypothesized that DNA hypomethylation in the caspase-3 gene promoter and/or enhancers is likely the mechanism for elevated caspase-3 gene transcription in *Uhrf1*^*ki/ki*^*/Apc*^*Min*/+^ mice. To test this hypothesis, we first identified the mouse intestinal caspase-3 enhancer regions according to the overlapping peaks of enhancer H3K4me1 and H3K27ac epigenetic marks in UCSC genome browser (Fig. 5B). We also downloaded DNA-methylation data of mouse caspase-3 locus from methbank (https://bigd.big.ac.cn/methbank/jbrowse) (Fig. 5B). This data mining showed the caspase-3 gene promoter as a CpG island with extremely low DNA methylation (Fig. 5B). In addition, six enhancer regions upstream of the caspase-3 promoter could be assigned according to overlapped H3K4me1 and H3K27ac peaks. Among these six putative enhancers, four (putative enhancers 2–5) were relatively poor in the CpG content and thus were not analyzed further. We prepared small intestinal epithelial cells from *Uhrf1*^+/+^ and *Uhrf1*^*ki/ki*^ mice in both control and *Apc*^*min*/+^ background and carried out high-throughput bisulfite sequencing analysis for enhancer 1, enhancer 6, and the promoter, and the results were shown in Fig. 5C. Consistent with the data in methbank, we found that the promoter CpG island was extremely low in methylation and there was no significant difference between the small intestinal samples from *Uhrf1*^+/+^, *Uhrf1*^*ki/ki*^, *Uhrf1*^*ki/ki*^*/Apc*^*Min*/+^, and *Uhrf1*^+/+^*/Apc*^*Min*/+^ mice. Comparison between *Uhrf1*^+/+^ and *Uhrf1*^*ki/ki*^ in control background revealed a modest reduction of DNA methylation across enhancers 1 and 6 (Fig. 5C, lower panel), consistent with the global DNA hypomethylation phenotype in *Uhrf1*^*ki/ki*^ mice. Interestingly, a more significant reduction of DNA methylation was observed for both enhancer 1 and enhancer 6 between *Uhrf1*^*ki/ki*^*/Apc*^*Min*/+^ and *Uhrf1*^+/+^*/Apc*^*Min*/+^ mice, especially at two CpG sites within the enhancer 6, which exhibit a near complete demethylation. Notably, sequence analysis revealed enhancers 1 and 6 contain multiple putative binding sites for transcription factors TCF1/2, AP-1 and c-Jun, the transcription factors that are typically activated by canonical and noncanonical Wnt pathways^55–57^. We thus proposed that loss of *Apc*-induced activation of Wnt pathways activate transcription factors TCF1/2, AP-1, and c-jun, which selectively activate caspase-3 gene transcription in the small intestinal epithelium of *Uhrf1*^*ki/ki*^*/Apc*^*Min*/+^ mice by binding more efficiently to hypomethylated caspase-3 enhancers. In addition, the binding of AP-1/c-jun may further promote DNA demethylation.

## Discussion

Virtually all human cancers display aberrant patterns of DNA methylation, including global hypomethylation punctuated by regional hypermethylation of gene promoters that often lead to tumor-suppressor gene silencing^1–5^. Global DNA hypomethylation occurs early in tumorigenesis, implying a driving role in oncogenesis. By treating *Dnmt1*^+/–^*Apc*^*Min*/+^ mice weekly with a low dose of 5-azaC, Laird et al. first demonstrated that 5-azaC-induced DNA hypomethylation almost completely suppresses intestinal adenomas in *Dnmt1*^*+/*–^*Apc*^*Min*/+^ mice^20^. Subsequent studies with various *Dnmt1* hypomorphic mice collectively demonstrated that genomic DNA hypomethylation has dual roles in tumorigenesis, strongly suppressing intestinal adenomas^15,18^, squamous tongue, and esophagus carcinogenesis^58^, and overall tumorigenesis in prostate cancer^14^, but promoting T-cell lymphoma^11^, intestinal microadenoma^15^, and early stages of prostate cancer and liver cancer^14,15^. Global DNA hypomethylation is thus proposed to promote tumorigenesis in cell types relying on genome instability and mutation for carcinogenesis and suppress tumorigenesis in cells where DNA-methylation-dependent silencing of tumor-suppressor genes is required. In this study, we used a *Uhrf1-TTD-KI* mouse model to investigate the role and mechanism by which DNA hypomethylation affects intestinal tumorigenesis (Fig. 1). We show that a moderate DNA hypomethylation in this mouse model is sufficient to suppress intestinal tumorigenesis (Figs. 2 and 3). Extensive mechanistic studies link the suppressed intestinal tumorigenesis to activation of caspase-3 gene expression and activity and increased apoptosis (Fig. 4 and Supplementary Figs. 3–6). The activation of caspase-3 expression is likely due to DNA hypomethylation in caspase-3 enhancers (Fig. 5). However, it remains to be tested if the activation of caspase-3 enhancers is casual to activation of apoptosis and tumor suppression observed in Uhrf1ki/ki DNA hypomethylation mouse model. Future studies are needed to elucidate further the mechanisms by which DNA hypomethylation suppresses intestinal tumorigenesis.

Previous studies of global DNA hypomethylation on tumorigenesis in mice all rely on *Dnmt1* hypomorphic mouse models with a variable but substantial reduction of Dnmt1 protein. As Dnmt1 is known to interact with various proteins and thus may possess DNA-methylation-independent functions^59–61^, additional *Dnmt1*-independent models would be valuable for further defining and investigating the diverse roles of DNA hypomethylation in cancers. The *Uhrf1-TTD-KI* mice used in this study are an ideal and complementary model for the following reasons. First, Uhrf1 is an essential accessory protein for Dnmt1-catalyzed DNA maintenance methylation, and the *Uhrf1-TTD-KI* mutation drives a moderate DNA hypomethylation in mice without affecting Dnmt1 protein level (Fig. 1). Second, unlike some *Dnmt1* hypomorphic mice, *Uhrf1-TTD-KI* mice display no observable phenotypes in development and postnatal growth (Fig. 1). Although a direct comparison between *Dnmt1* hypomorphic mice and *Uhrf1-TTD-KI* mice in the levels of DNA hypomethylation is not yet available, the latter mice are likely less severe in DNA hypomethylation, as only ~10% reduction of global DNA methylation was detected in various tissues that we have tested (Fig. 1) and activation of interferon pathway is not observed (Supplementary Fig. 6). Nevertheless, by using chemical-induced and *Apc*^Min/+^ intestinal tumor models, we demonstrated that the moderate DNA hypomethylation caused by *Uhrf1-TTD* mutation is sufficient to markedly suppress intestinal tumorigenesis. Interestingly, unlike studies with *Dnmt1* hypomorphic mice, both micro- and macroadenoma were suppressed in *Uhrf1-TTD-KI* mice (Fig. 3). This discrepancy in microadenoma formation is likely complicated and warrants for future studies. One possibility is that the degree of DNA hypomethylation may dictate whether it promotes or suppresses intestinal microadenoma. In this regard, a conditional but complete deletion of *Dnmt1* in adult *Apc*^Min/+^ mice was shown to accelerate rather than suppress intestinal adenoma initiation^62^. Alternatively, the enhanced microadenoma observed in *Dnmt1* hypomorphic mice could be due to reduced Dnmt1 protein level and not a result of DNA hypomethylation.

In defining the molecular mechanism(s) underlying suppressed intestinal tumorigenesis in *Uhrf1-TTD-KI* mice, we found no evidence for differences in *Apc* LOH, activation of Wnt and Hippo pathways, and cell proliferation (Supplementary Figs. 3–5). Instead, we detected significantly increased apoptosis in intestinal tissues of *Uhrf1*^*ki/ki*^*/Apc*^*Min*/+^ mice as compared to *Uhrf1*^+/+^*/Apc*^*Min*/+^ mice (Fig. 4). Consistent with elevated apoptosis, we detected significantly increased levels of activated caspase-3 as well as full-length caspase proteins in intestinal tissues of *Uhrf1*^*ki/ki*^*/Apc*^*Min*/+^ mice vs. *Uhrf1*^+/+^*/Apc*^*Min*/+^ mice. Notably, elevated caspase-3 expression and activation were observed in intestinal but not liver tissues, consistent with a previous report showing DNA hypomethylation suppresses intestinal tumor formation but promotes liver carcinogenesis^15^. It is also noteworthy that the caspase-3 levels in tumors were actually reduced when compared to normal intestinal tissues of *Uhrf1*^*ki/ki*^*/Apc*^*Min*/+^ mice, consistent with the notion that apoptosis is generally suppressed during tumorigenesis. The facts that caspase-3 and apoptosis are markedly elevated in normal intestinal tissues of *Uhrf1*^*ki/ki*^*/Apc*^*Min*/+^ mice are consistent with our observation that both micro- and macro-adenomas are suppressed in *Uhrf1*^*ki/ki*^*/Apc*^*Min*/+^ mice. Thus, elevated expression and activation of caspase-3 and the resulting elevated apoptosis are likely a mechanism for intestinal tumor suppression by DNA hypomethylation in *Uhrf1*^*ki/ki*^*/Apc*^*Min*/+^ mice. However, activation of the Casp3 enhancer may not be the sole cause of reduced tumorigenesis. Despite elevated expression of caspase 3, it still could be an effect, rather than a cause, of the increased apoptosis. In addition, it remains to be tested if elevated expression and activation of caspses-3 is sufficient to suppress intestinal tumorigenesis. Furthermore, we could not exclude that other possibilities including activation of other components in the apoptosis pathway, activation of other cell death pathway or impaired H3K9me2/3 binding of UHRF1 may also contribute to the tumor suppression phenotype.

**Fig. 6:**
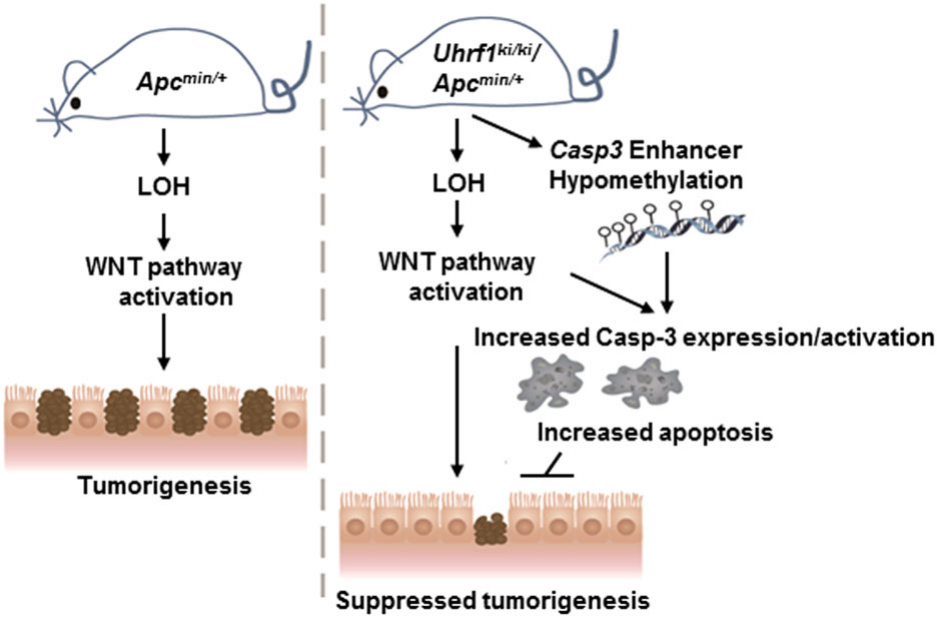
DNA hypomethylation and Wnt pathway cooperatively activate caspase-3 expression to suppress intestinal tumorigenesis. Working model illustrates how DNA hypomethylation suppresses intestinal tumorigenesis by promoting caspase-3 expression and apoptosis. In *Uhrf1*^*ki/ki*^ mice or *Apc*^*min*/+^ mice, DNA hypomethylation or activation of Wnt pathway alone is not sufficient to significantly enhance caspase-3 gene expression. However, DNA hypomethylation caused by *Uhrf1-TTD-KI* mutation can promote caspase-3 transcriptional activation by Wnt canonical and noncanonical pathway-activated transcription factors such as TCF1/2, AP-1, and c-jun and β-catenin, which in turn leads to elevated apoptosis and tumor suppression.

We found that enhancer DNA hypomethylation rather than promoter demethylation is associated with increased caspase-3 gene expression. Promoter hypermethylation is widely believed to account for epigenetic silencing of tumor-suppressor genes occurring during tumorigenesis. Our extensive DNA-methylation analysis revealed that the CpG island caspase-3 promoter was unmethylated in *Uhrf1*^*ki/ki*^*/Apc*^*Min*/+^ and *Uhrf1*^+/+^*/Apc*^*Min*/+^ mice. However, we found substantially reduced DNA methylation in two putative caspase-3 enhancer regions (Fig. 5C). This observation points to the possibility that enhancer DNA hypomethylation is likely to contribute to elevated caspase-3 expression and apoptosis in *Uhrf1*^*ki/ki*^*/Apc*^*Min*/+^ mice. This finding is also consistent with recent genome-wide DNA-methylation studies showing widespread DNA-methylation changes in enhancer regions under physiologic and pathologic conditions^63–65^. Notably, there is no significant change in caspase-3 expression in the intestinal tissues between *Uhrf1*^*ki/ki*^ and *Uhrf1*^+/+^ mice or between *Apc*^*Min*/+^ and *Apc*^+/+^ mice, suggesting a combinatorial effect of DNA hypomethylation and activation of Wnt signaling pathways in driving caspase-3 expression and apoptosis. In support of this hypothesis, bisulfite sequencing analysis revealed a less significant reduction of DNA methylation in caspase-3 enhancers in *Uhrf1*^*ki/ki*^ mice as compared to *Uhrf1*^+/+^ mice (Fig. 5C, lower panel). These results suggest a working model in Fig. 6, in which activation of Wnt signaling pathways leads to activation of transcription factors TCF1/2, AP-1, and c-Jun, the downstream effectors of Wnt canonical and noncanonical pathways. The binding of one or more of these transcription factors may initiate further DNA demethylation of caspase-3 enhancer regions and activation of caspase-3 expression, which contributes to increased apoptosis and tumor suppression in intestinal tissues of *Uhrf1*^*ki/ki*^*/Apc*^*Min*/+^ mice.

In sum, we present a novel *Uhrf1-TTD-KI* mouse model for investigating global DNA hypomethylation in tumorigenesis. Using this mouse model, we demonstrated that moderate global DNA hypomethylation is sufficient to markedly suppress intestinal tumorigenesis and provided evidence that enhanced caspase-3 expression driven by enhancer DNA hypomethylation and activation of Wnt pathways may lead to elevated apoptosis and suppression of intestinal tumorigenesis (Fig. 6). Targeting epigenetic modifications including DNA methylation has recently emerged as an important therapeutic approach for colorectal cancers^66,67^. Our finding that moderate DNA hypomethylation is sufficient to markedly suppress intestinal tumorigenesis suggests potentially using low doses of DNA-methylation inhibitors as a means of colorectal cancer prevention.

## Material and methods

### Animals

All mice were maintained in the Animal Research Center of East China Normal University under pathogen-free conditions at 22 ± 2 °C. All mice were maintained in the C57Bl/6J genetic background. All animal experiments were performed in accordance with guidelines and regulations drafted by the Association for Assessment and Accreditation of Laboratory Animal Care in Shanghai (Ethical number: m20141202 and m20191206). Intestinal tumors were counted by using stereomicroscope without gender selection. Littermate genotype controls were used in all experiments. The animal experiments were repeated twice at least, and the numbers of animals (at least three for each group) in the experimental groups were described in the figure legends.

### Antibodies

The following primary antibodies were used in this study: c-Myc (Abcam, ab84132), GAPDH (Absci, AB41549), ACTIN (Huaan, M1210-2), UHRF1 (homemade), DNMT3A (Santa Cruz, sc-20703), DNMT1 (Huaan, ET1702-77), H3K9me3 (homemade), H3 (Abcam, ab1791), β-catenin (Huaan, ER080), Ki67 (Huaan, ER1706-46), AXIN1 (CST, 2087), GSK-3β (Klean AB, P1018 48), Caspase-3 (CST, 9662), cleaved-Caspase-3 (CST, 9664), and PARP (CST, 9532).

### *Uhrf1*-TTD-KI, *Apc*^*min*/+^ and compound mouse models

*Uhrf1-TTD-KI* mice were described previously^39^. To generate compound *Uhrf1-TTD-KI* and *Apc*^min/+^ mice, *Uhrf1*^ki/ki^ mice were mated with *Apc*^min/+^ to produce F1 generations. Then male *Uhrf1*^ki/+^*/Apc*^min/+^ were selected to cross with female *Uhrf1*^ki/+^*/Apc*^+/+^ to obtain the F2 generation with four different genotypes: *Uhrf1*^ki/ki^*/Apc*^min/+^, *Uhrf1*^+/+^*/Apc*^min/+^, *Uhrf1*^ki/ki^*/Apc*^+/+^, and *Uhrf1*^+/+^*/Apc*^+/+^ mice. The mice were genotyped by DNA sequencing or PCR of genomic DNA derived from mouse toe with specific primers as listed in Supplementary Table 3. The mice were sacrificed at 20 weeks of age for examining intestinal and colorectal tumors.

### AOM/DSS model

Induction of colorectal tumors in wild-type and *Uhrf1*^ki/ki^ mice by AOM/DSS was carried out essentially as described^42^. In brief, mice (2.5-month old) were treated with a single intraperitoneal dose (10 mg/kg of body weight) of AOM (sigma, A5486) followed by three 5-day cycles of 2% DSS in drinking water, with each cycle followed by a 2-week recovery. The mice were sacrificed at 90 days from initial AOM injection for analyzing colorectal tumors by both stereomicroscope and tissue section staining. The mice were sacrificed at 5.5-month old.

### Paraffin tissue section, H&E staining, and Alcian blue staining

Small intestine or colon tissues were dissected and removed from the sacrificed mice. The lumens of the intestine tissues were carefully flushed with ice-cold PBS using a 50-ml syringe and intragastric needle. Cleaned intestine tissues were rolled-up and fixed in 4% paraformaldehyde, dehydrated and embedded into paraffin. Five micrometer sections were cut and stained with H&E or 1% Alcian blue (pH 2.5). The alcian blue staining should be counterstained with nuclear fast red.

### Immunohistochemistry (IHC)

IHC staining were performed using paraffin-embedded tissues. Antigen retrieval was performed in citrate buffer (1.8% 0.1-M citric acid, 8.2% 0.1-M sodium citrate) for 20 min at 100 °C; sections were washed in PBS, incubated for 10 min in 3% H_2_O_2_ (for IHC), blocked in goat serum from kit (Vector PK-4001), incubated for 16 h at 4 °C in primary antibody, and washed in PBS. Secondary antibody reaction was performed using kits (Vector PK-4001 and SK-4100, VECTOR) according to manufacturer’s instruction. The slides were counterstained with hematoxylin and embedded using Entellan (Merck Millipore). The following antibodies were used: anti-mouse β-catenin (Huaan, ER080, 1:500), anti-mouse Ki67 (Huaan, ER1706-46, 1:100), anti-mouse cleaved-Caspase-3 (Cell Signaling Technology, 9664, 1:100). Images were taken using Olympus BX53 microscope.

### BrdU immunofluorescence staining

Mice were intraperitoneally injected with BrdU (Sigma, 100 mg/kg) 5 h or 50 h before sacrificed. BrdU immunofluorescence staining was performed using paraffin-embedded tissues. Antigen retrieval was performed in citrate buffer (1.8% 0.1-M citric acid, 8.2% 0.1-M sodium citrate) for 20 min at 100 °C; sections were washed in PBS, incubated for 10 min in 3% H_2_O_2_, blocked in goat serum from kit (Vector PK-4001), incubated for 16 h at 4 °C with anti-BrdU (Sigma-Aldrich, B928, 1:50). Then, sections were incubated with Alexa Fluor 488 goat anti-mouse IgG (Life Technologies) and DAPI (1 μg/ml, Sigma-Aldrich) for 1 h at room temperature, washed, and embedded using FluorSave (Merck Millipore). Images were taken using Leica DM4000 microscope.

### TUNEL assay

TUNEL assay was performed by the TUNEL Apoptosis Detection kit (Yeasen biotech, 40308ES20) according to manufacturer’s instruction. The slides were visualized by fluorescence confocal microscope (Leica SP8).

### Intestinal epithelium isolation and polyp microdissection

Isolation of small intestinal epithelium for analysis was performed essentially as previously described^68^. Polyps were microdissected from small intestine under ×10–20 magnification on a dissecting microscope, and were homogenized in the RIPA buffer for Western blotting lysis and Total Trizol for RNA extraction or genomic DNA extraction.

### Western blotting analysis

Small intestinal epithelial cells or tumor tissues that were first broken up by a tissue grinding apparatus were lysed with the RIPA buffer on ice for 2 h. After centrifugation at 12,000 rpm for 10 min at 4 °C, the clean extracts were obtained and subjected to Western blotting analysis essentially as described^69^. The immunereactive proteins were detected by the Odyssey laser digital imaging system.

### Measurement of 5mC by HPLC

Genomic DNA was prepared from intestinal epithelium, mouse tissues, or tumors and dissolved in ddH_2_O. The measurement of 5mC levels was performed as described^39^.

### qRT-PCR analysis

Total RNAs were extracted from the intestinal epithelium cells or microdissected fresh tumor tissues by using the Total Trizol extraction from TOROIVD (A211-01). cDNAs were synthesized using the reverse transcription kit TOYOBO (FSQ-301). Subsequent PCRs were performed using the TOROGreen^®^ qPCR Master Mix (TOROIVD, QST-100) on the QuantStudio 3 Real-Time PCR System (Applied Biosystems) using the comparative Ct quantitation method. Results were normalized to actin B *mRNA* levels. PCR protocol was as follows: initial denaturing at 95 °C for 5 min, 40 cycles of 95 °C for 20 s, 60 °C for 30 s, and 72 °C for 30 s, followed by final extension at 72 °C for 5 min. Melt curve analysis was performed on a QuantStudio 3 Real-Time PCR System (Applied Biosystems) coupled with QuantStudio™ Design & Analysis Software. All primer sequences for qRT-PCR analyses are listed in Supplementary Table 3.

### Bisulfite sequencing

Bisulfite conversion was performed using the EZ DNA Methylation-GoldTM Kit (ZYMO Research) according to manufacturer’s instruction manual. Bisulfite-converted DNA was used in PCR amplification by TaKaRa Ex Taq HS (Takara, RR006A). The PCR products were gel-purified and used for Illumina^®^ (ND102-0102) sequencing. The levels of methylation at each CpG sites were calculated based on large number (up to thousands) of sequenced DNA as described^69^. The primers for PCR amplification are listed in Supplementary Table 3.

### RNA-sequencing analysis

Total RNAs were prepared from the small intestinal epithelial cells of three pairs of *Uhrf1*^*ki/ki*^ and *Uhrf1*^+/+^ littermates. RNA-seq analysis was performed by Berry Genomics Co., Ltd. (Beijing, China). Gene expression analysis was performed as described^70^.

### Statistical analysis

All statistical analysis was performed via GraphPad Prism 5.0 software. All values are depicted as the mean ± SEM. Student *t* test was used in analyzing the statistical significance of experiments comparing two groups. All Western blotting experiments were done at least for three times. Overall survival of different groups of mice was evaluated by the Kaplan–Meier survival analysis and the log-rank test. Differences were considered statistically significant at *p* < 0.05. The positive signals of β-catenin, cleaved-caspase-3, Ki67, and PCNA were analyzed by using ImageJ Analysis.

## Supplementary information

Supplementary Figure S1-6

Supplementary Table S1

Supplementary Table S2

Supplementary Table S3

## Data Availability

Murine small intestinal caspase-3 H3K4me1 and H3K27ac genomic profiles were downloaded from data in UCSC genome browser: http://genome.ucsc.edu/cgi-bin/hgTracks?db=mm9&lastVirtModeType=default&lastVirtModeExtraState=&virtModeType=default&virtMode=0&nonVirtPosition=&position=chr8%3A47681543-47745322&hgsid=920520527_JiUBTKq6u7USrIL8Z6vC3aEnNYBR.
