# Supplementary material for: Moderate DNA hypomethylation suppresses intestinal tumorigenesis by promoting caspase-3 expression and apoptosis
Source: Oncogenesis. 2021 May 4;10(5):38. doi: 10.1038/s41389-021-00328-9 (PMC8096944; doi:10.1038/s41389-021-00328-9)
Supplement: Supplementary file 1 — Supplementary Figure S1-6 [file 41389_2021_328_MOESM1_ESM.pdf]

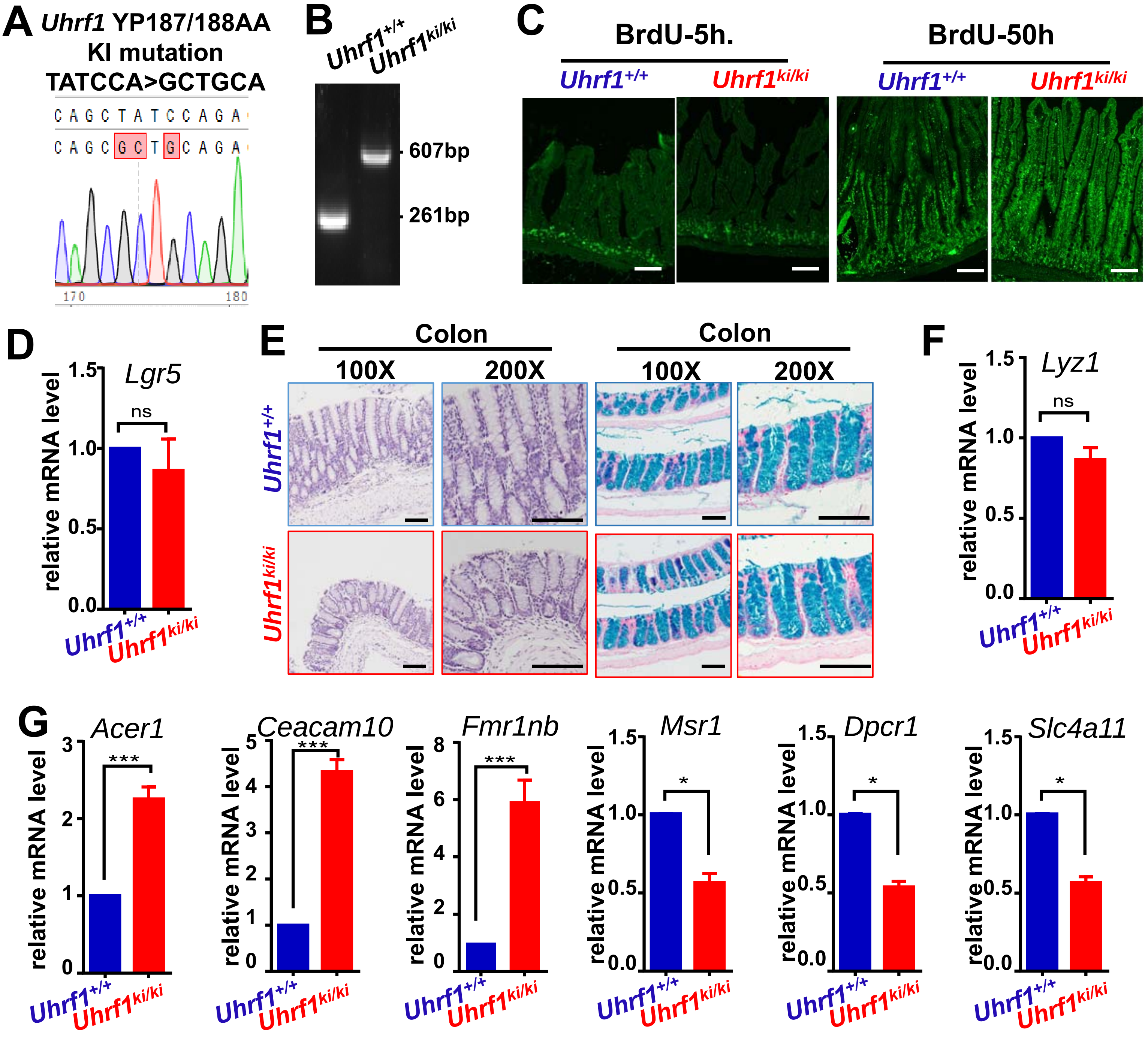


**Supplementary Figure S1. Characterization of *Uhrf1*-TTD-KI (*Uhrf1*$^{ki/ki}$) mutant mice.** (**A**) Genotyping by DNA sequencing. The representative sequencing data show GCTGCA sequence that encodes Alanine 187 and Alanine 188 in homozygous *Uhrf1*$^{ki/ki}$ mutant mice. The corresponding sequence in *Uhrf1*$^{+/+}$ mouse is TATCCA, which encodes Tyrosine 187 and Proline 188. (**B**) Genotyping by PCR-based DNA amplification. *Uhrf1*$^{+/+}$ and *Uhrf1*$^{ki/ki}$ mutant mice gave rise to a 261bp and a 607bp PCR product, respectively. (**C**) Representative immunofluorescence staining for incorporated BrdU in small intestinal sections from *Uhrf1*$^{+/+}$ and *Uhrf1*$^{ki/ki}$ mutant mice. Scale bars, 100 μm. (**D**) qRT-PCR analysis showing similar levels of stem cell marker Lgr5 in *Uhrf1*$^{+/+}$ and *Uhrf1*$^{ki/ki}$ mutant mice. ns, no significant with p >0.05. (**E**) HE staining (left) and alcian blue staining (right) showing similar colon structure and numbers of Goblet cells in colon tissues from *Uhrf1*$^{+/+}$ and *Uhrf1*$^{ki/ki}$ mutant mice. Shown were representative results from 8-week mice. Scale bar, 100μm. (**F**) qRT-PCR analysis showing similar levels of Paneth cell marker Lyz1 in *Uhrf1*$^{+/+}$ and *Uhrf1*$^{ki/ki}$ mutant mice. (**G**) Verification of differential gene expression by qRT-PCR analysis. The differentially expressed genes were identified by RNA-seq analysis. For (**D**), (**F**) and (**G**), the level of mRNA in the *Uhrf1*$^{+/+}$mice was set as 1 for each gene. *Uhrf1*$^{+/+}$, n=3; *Uhrf1*$^{ki/ki}$, n=3. Error bars, S.E.***p < 0.001; * p < 0.05.

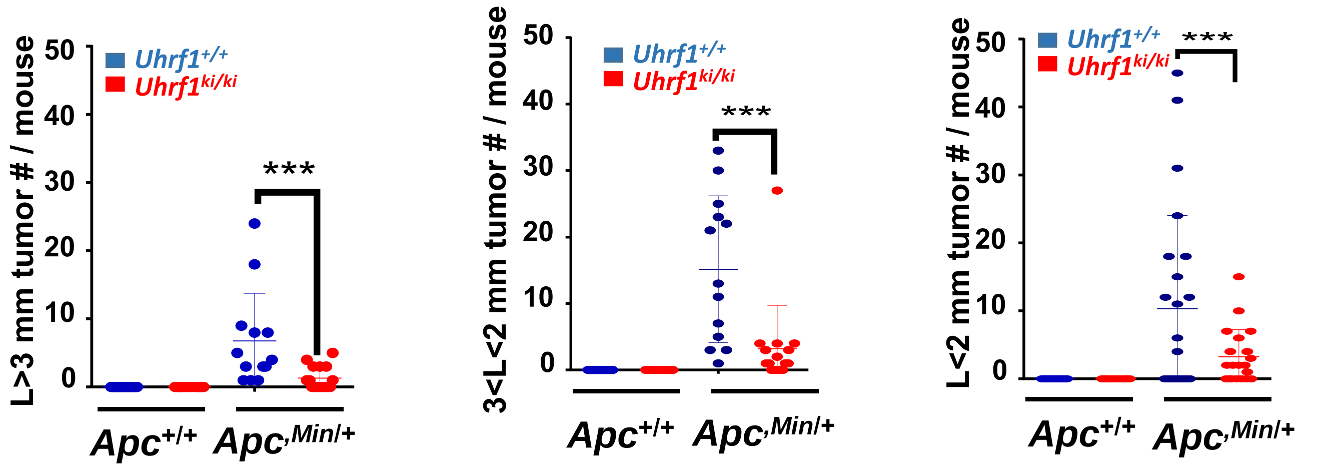


**Supplementary Figure S2. Intestinal tumorigenesis is substantially suppressed in $\textit{Uhrf1}^{\ ki/ki}/\textit{Apc}^{min/+}$ mice.** Tumors were classified according to sizes into groups with diameter > 3 mm, 3 mm < L < 2 mm, and L < 2 mm. The tumors were then plotted according to the numbers of tumors per mouse. The numbers of mice analyzed: $\textit{Uhrf1}^{+/+}/\textit{Apc}^{+/+}$, n=21; $\textit{Uhrf1}^{ki/ki}/\textit{Apc}^{+/+}$, n=16; $\textit{Uhrf1}^{+/+}/\textit{Apc}^{min/+}$, n=23; *and* $\textit{Uhrf1}^{\ ki/ki}/\textit{Apc}^{min/+}$, n=20. Error bars, S.E. ***p < 0.001.

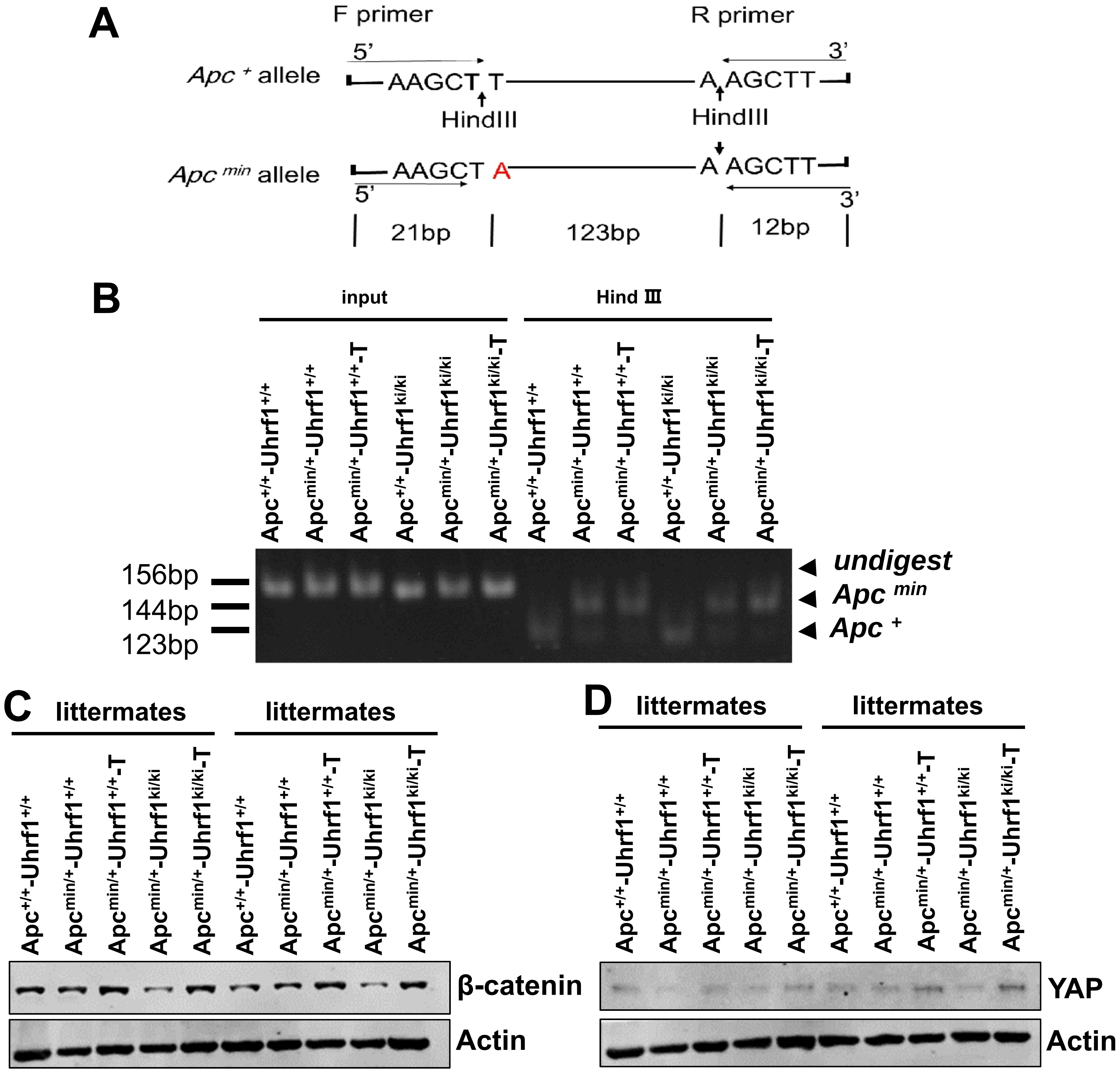


**Supplementary Figure S3. DNA hypomethylation caused by *Uhrf1-TTD-KI* mutation has no effect on *Apc* LOH and activation of Wnt and Hippo pathways in *Apc*$^{min/+}$ mice.** (**A**) The schematic diagram of PCR-based detection of *Apc* LOH. The *Apc*$^{+}$ and *Apc*$^{min}$ alleles differ in HindⅢ digestion, with a 123 bp product for *Apc*$^{+}$ allele and 144 bp product for *Apc*$^{min}$ allele. (**B**) *Apc*$^{+}$ LOH was detected in small intestinal tissues and tumors from both *Uhrf1*$^{+/+}$/*Apc*$^{min/+}$; *Uhrf1* $^{ki/ki}$/ *Apc*$^{min/+}$ mice. PCR-based assay of *Apc* locus was performed with genomic DNA from small intestinal epithelium cells or microdissected tumors from *Uhrf1*$^{+/+}$/*Apc*$^{+/+}$, *Uhrf1* $^{ki/ki}$/ *Apc*$^{+/+}$, *Uhrf1*$^{+/+}$/*Apc*$^{min/+}$, and *Uhrf1* $^{ki/ki}$/ *Apc*$^{min/+}$ mice. (**C-D**) Western blot analysis showing the levels of β-catenin (**C**) and YAP (**D**) in small intestinal epithelium cells from *Apc*$^{+/+}$-*Uhrf1*$^{+/+}$, *Apc*$^{min/+}$-*Uhrf1*$^{+/+}$ and *Apc*$^{min/+}$-*Uhrf1*$^{ki/ki}$ mice, or microdissected tumors from *Apc*$^{min/+}$-*Uhrf1*$^{+/+}$ and *Apc*$^{+/+}$-*Uhrf1*$^{ki/ki}$ mice. Note that tumors from both *Apc*$^{min/+}$-*Uhrf1*$^{+/+}$ and *Apc*$^{min/+}$-*Uhrf1*$^{ki/ki}$ mice have elevated levels of β-catenin and YAP proteins. In **B-D**, *Apc*$^{min/+}$-*Uhrf1*$^{+/+}$-T and *Apc*$^{min/+}$-*Uhrf1*$^{ki/ki}$-T represent the tumor samples from *Apc*$^{min/+}$-*Uhrf1*$^{+/+}$ and *Apc*$^{min/+}$-*Uhrf1*$^{ki/ki}$ mice, respectively.

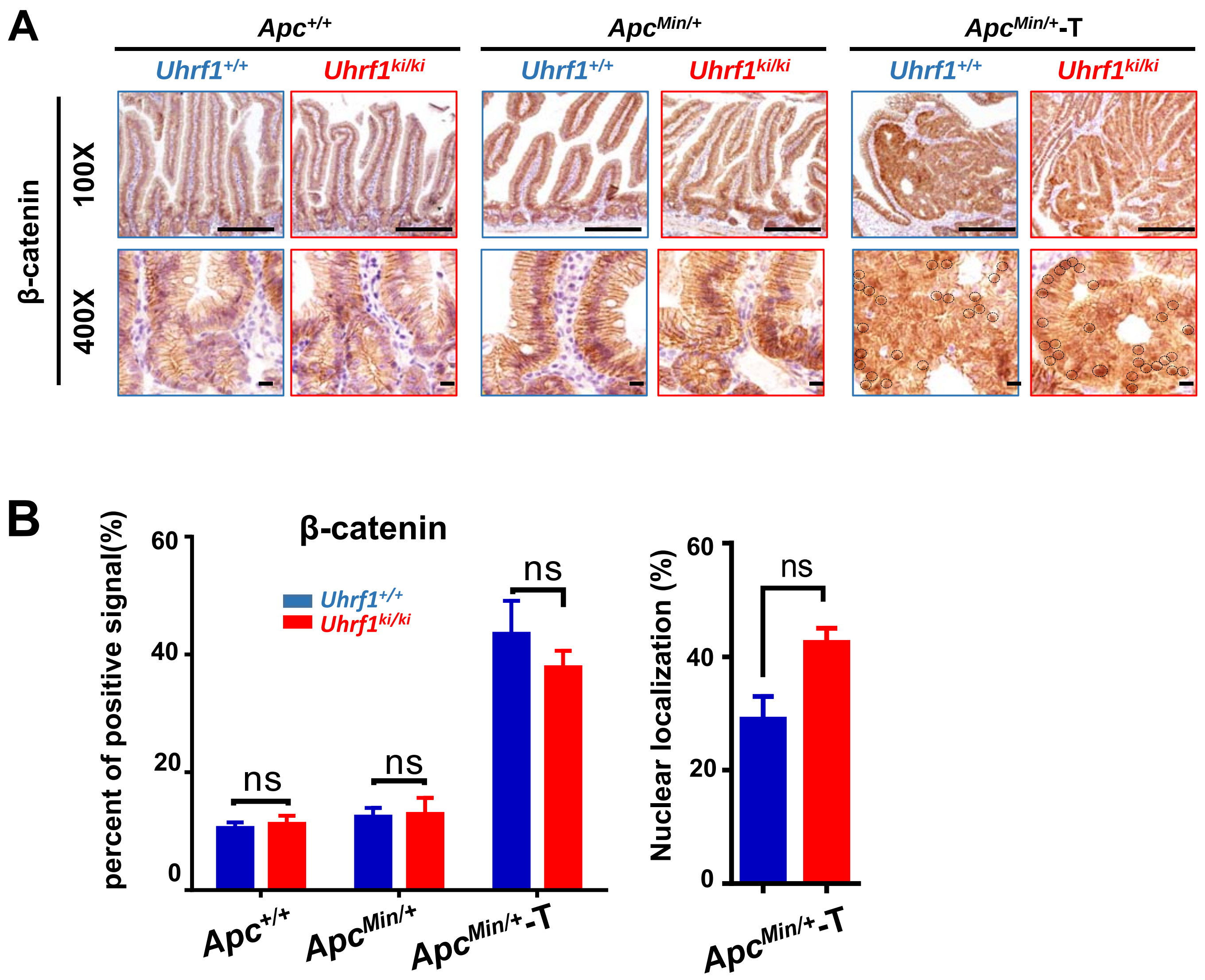


**Supplementary Figure S4. Analysis of β-catenin expression and nuclear localization by immunohistochemistry.** (**A**) Immunohistochemistry analysis showing β-catenin level and subcellular localization in small intestinal tissues from $Uhrf1^{+/+}/Apc^{+/+}$, $Uhrf1^{ki/ki}/Apc^{+/+}$, $Uhrf1^{+/+}/Apc^{min/+}$, and $Uhrf1^{ki/ki}/Apc^{min/+}$ mice. Intestinal tissue sections without or with tissues were processed for IHC using anti- β-catenin antibody. Scale bar, 50µm. Note β-catenin nuclear localization was observed in intestinal tumors from both $Uhrf1^{+/+}/Apc^{min/+}$ and $Uhrf1^{ki/ki}/Apc^{min/+}$ mice. (**B**) Quantitative analysis of β-catenin positive cells and cells with β-catenin nuclear localization. The IHC data in (**A**) were analyzed by ImageJ about the percentage of β-catenin immunohistochemistry signal positive cells (left) and the percentage of nuclear localized β-catenin cells (right). $Uhrf1^{+/+}/Apc^{+/+}$, n=3; $Uhrf1^{ki/ki}/Apc^{+/+}$, n=3; $Uhrf1^{+/+}/Apc^{min/+}$, n=3; $Uhrf1^{ki/ki}/Apc^{min/+}$, n=3; $Uhrf1^{+/+}/Apc^{min/+}$-T, n=3; and $Uhrf1^{ki/ki}/Apc^{min/+}$-T, n=3. Error bars, S.E. ns, $p > 0.05$. $Apc^{+/+}$, normal intestinal samples from $Apc^{+/+}$ mice; $Apc^{min/+}$, non-tumor intestinal samples from $Apc^{min/+}$ mice; and $Apc^{min/+}$-T, tumor samples from $Apc^{min/+}$ mice.

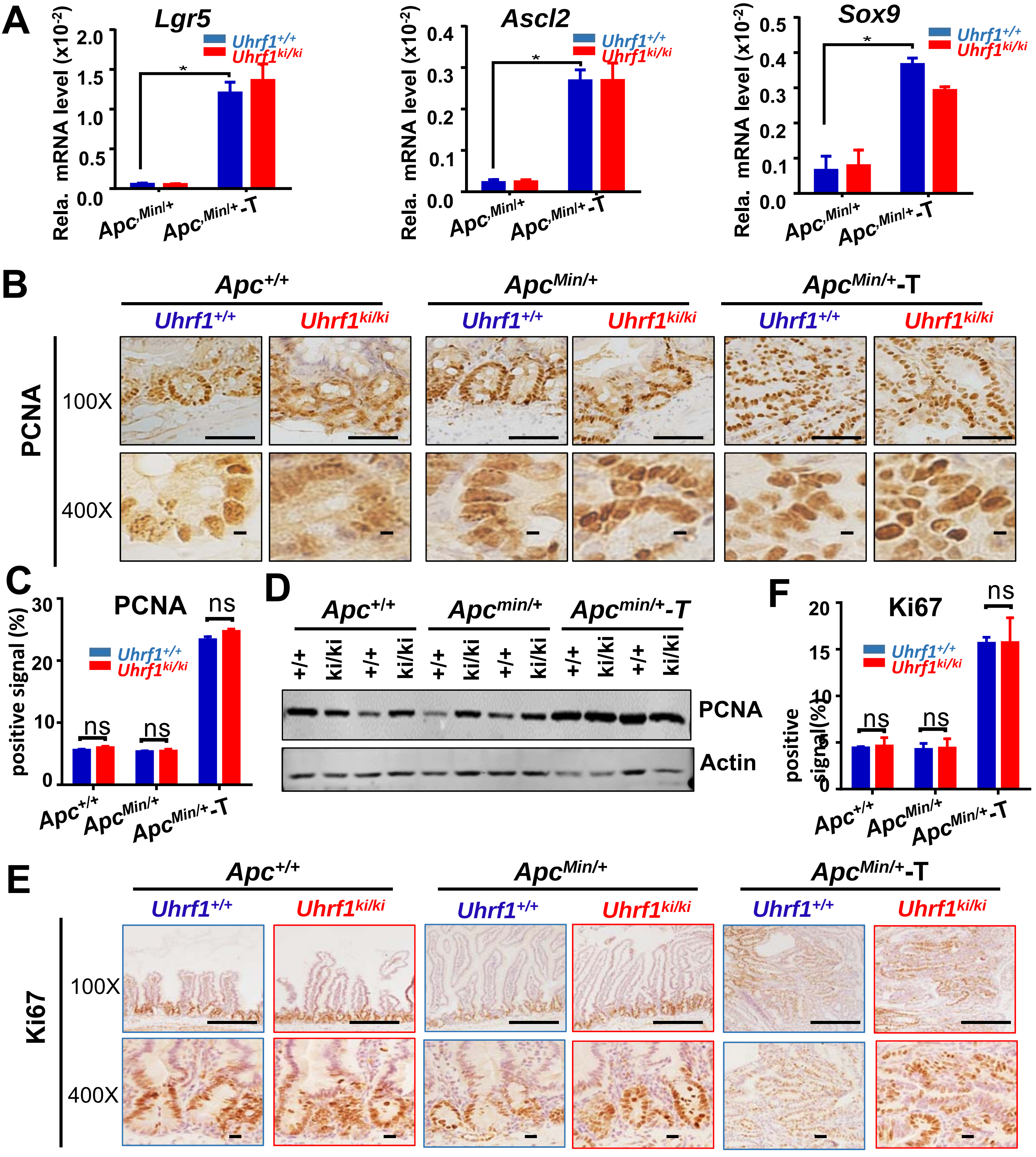


**Supplementary Figure S5. DNA hypomethylation caused by *Uhrf1-TTD-KI* mutation does no affect tumor cell proliferation. (A)** qRT-PCR analysis showing the relative expression levels of intestinal stem cell markers *Lgr5, Ascl2* and *Sox9* in intestinal epithelium cells or microdissected tumors from mice as indicated. $Uhrf1^{+/+}/Apc^{min/+}$, n=8; $Uhrf1^{ki/ki}/Apc^{min/+}$, n=8; $Uhrf1^{+/+}/Apc^{min/+}$-T, n=3; and $Uhrf1^{ki/ki}/Apc^{min/+}$-T, n=3. Error bars, S.E. $*p < 0.05$. (**B**) IHC analysis showing the levels of PCNA in small intestinal tissues or tumors from different mice. Scale bar, 50μm. (**C**) Quantitative results of PCNA positive cells based on IHC results in (**B**). N=3 for each samples. (**D**) Western blot results showing the levels of PCNA proteins in small intestinal epithelium cells or microdissected tumors from various mice as indicated. (**E**) IHC analysis showing the levels of Ki67 in small intestinal tissues or tumors from various mice. Scale bar, 50μm. (**F**) Quantitative results of Ki67 positive cells based on IHC results in (**E**). N=3 for each samples.

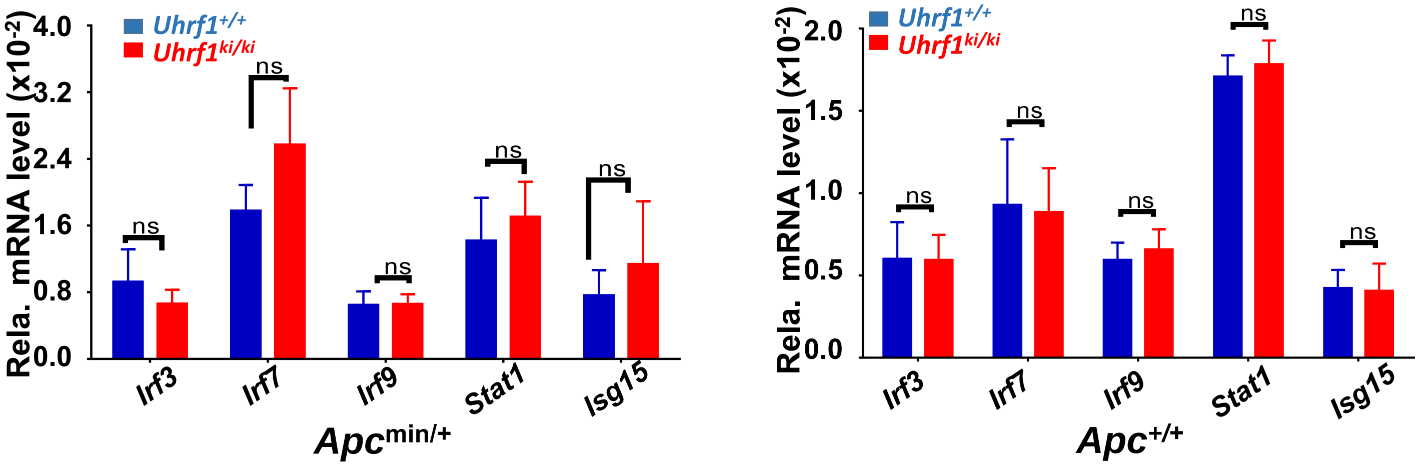


**Supplementary Figure S6. DNA hypomethylation caused by *Uhrf1-TTD-KI* mutation does no lead to activation of interferon pathway in *Apc*$^{min/+}$ mice.** Relative levels of key interferon pathway genes were examined by qRT-PCR analysis. The total RNAs were prepared from small intestinal epithelium cells derived from various mice as indicated. Note there is no activation of the interferon genes tested in *Uhrf1*$^{+/+}$/*Apc*$^{min/+}$ and *Uhrf1*$^{ki/ki}$/*Apc*$^{min/+}$ mice. The relative mRNA levels were normalized to *Actin*. *Uhrf1*$^{+/+}$/*Apc*$^{+/+}$, n=3; *Uhrf1* $^{ki/ki}$/*Apc*$^{+/+}$, n=3; *Uhrf1*$^{+/+}$/*Apc*$^{min/+}$, n=3 and *Uhrf1* $^{ki/ki}$/*Apc*$^{min/+}$, n=3). Error bars, S.E. ns (non significant).
