# Supplementary material for: Moderate DNA hypomethylation suppresses intestinal tumorigenesis by promoting caspase-3 expression and apoptosis
Source: Oncogenesis. 2021 May 4;10(5):38. doi: 10.1038/s41389-021-00328-9 (PMC8096944; doi:10.1038/s41389-021-00328-9)
Supplement: Supplementary file 2 — Supplementary Table S1 [file 41389_2021_328_MOESM2_ESM.pdf]

Supplementary Table S1. List of the differentially expressed genes in small intestinal epithelial cells in *Uhrf1-TTD-KI* mice

Genes	log2 fold change	P value
Clip4	4.764667553	0.01248376
4931440F15Rik	4.180333879	0.033518106
Lrrc74b	3.704264378	0.032425105
Hoxb9	3.699429736	0.021107486
4933411K16Rik	3.597217447	0.042580358
Nim1k	3.249400414	0.028717749
H2-Bl	2.876269373	0.022243668
Nkapl	2.664413628	0.044863898
Coro2b	2.645029996	0.025110488
Fmr1nb	2.102417884	1.19691E-08
Gspt2	2.001376122	0.039567993
5730507C01Rik	1.898920412	0.023913196
Ceacam10	1.879729582	6.62291E-08
Vmn2r26	1.876212018	0.024811762
Sertad4	1.788929771	0.047201418
Bcas1os2	1.766706994	0.041772364
4933417O13Rik	1.751101401	0.03840633
Nox1	1.657051572	0.004796505
Pak3	1.655783314	0.008440505
1600023N17Rik	1.586157372	0.03413574
Bglap3	1.56459427	0.000478524
Dnah9	1.550682777	0.047893897
Card9	1.460493087	0.016650396
H2-T22	1.435286226	0.04036295
Smarca5-ps	1.350545287	0.000990992
Hhipl2	1.333590382	0.022720861
Tdrd1	1.323915912	0.038695168
C530005A16Rik	1.302612407	0.038478791
H2-DMb1	1.283379456	0.049429247
Slfn4	1.241555542	0.001642839
Defb1	1.228017589	0.037434668
Hao2	1.194898499	0.029486665
Gm7030	1.143854904	0.007119925
Pxdn	1.127251459	0.011646315
Tlr2	1.078249583	0.019986666
Eps8l1	1.037246033	0.000444183
H2-Ab1	1.016100828	0.002729891
Slc15a2	1.007500048	0.022935839
Mptx1	0.990963756	0.01373014
Ubd	0.977361361	0.025255016
Nxf7	0.949138483	0.002990792

Acer1	0.936664815	4.95092E-05
Adgrg6	0.904427243	0.023349619
Fstl1	0.895095307	0.034938766
Mrc1	0.856169109	0.031156703
1700024P16Rik	0.851714775	0.020686533
Jdp2	0.851650341	0.049005029
Srd5a2	0.850778046	0.046528673
Cfap74	0.819382675	0.026493833
Sp140	0.800768732	0.026319991
Trerf1	0.776584984	0.040209234
Rps4l	0.748806169	0.00152649
H2-DMa	0.746357347	0.018798619
Sult1c2	0.704394111	0.040618666
Vwa1	0.670470857	0.01590952
Casc4	0.660137824	0.019406483
Kremen2	0.634982122	0.025091095
Trim5	0.616760142	0.030756017
Erdr1	0.605443067	0.027824589
H2-T-ps	0.602376035	0.028696832
Bcl3	-0.591142778	0.026675063
Ddah1	-0.632374936	0.030383601
Chst4	-0.635176321	0.016858101
Lyrm7	-0.683455298	0.045399866
Dennd1c	-0.720488709	0.006559267
Uhrf1	-0.78322004	0.000906011
Otop3	-0.81743022	0.015476801
Npl	-0.820104221	0.001634997
Ctse	-1.039011234	0.021270695
C3	-1.044537719	0.014787013
Cmtm8	-1.114043309	0.037034533
Ugt2b38	-1.163781719	0.029568231
Chst2	-1.226878273	0.019300606
Hist1h2be	-1.309259345	0.035335886
Hist1h4d	-1.458068966	0.044590106
Slc4a11	-1.694554489	0.008780572
Gpc3	-1.76538008	0.049646854
Gas1	-2.128515626	0.020041337
Catsperd	-2.236631922	0.018421948
Cpa3	-2.807071787	0.0354176
Dpcr1	-3.457431174	0.003002315
Psca	-3.59457733	0.044773317
Msr1	-3.803021419	0.030210537
Fgf13	-3.983362814	0.018170024

Csf3r	-4.026309948	0.046845353
Hoxaas3	-4.314727406	0.038680125
