# Supplementary material for: Moderate DNA hypomethylation suppresses intestinal tumorigenesis by promoting caspase-3 expression and apoptosis
Source: Oncogenesis. 2021 May 4;10(5):38. doi: 10.1038/s41389-021-00328-9 (PMC8096944; doi:10.1038/s41389-021-00328-9)
Supplement: Supplementary file 3 — Supplementary Table S2 [file 41389_2021_328_MOESM3_ESM.pdf]

**Supplementary Table S2. List of the differentially expressed transposable elements in small intestinal epithelial cells in *Uhrf1-TTD-KI* mice**

| TE | log2 fold change | P value |
|---|---|---|
| chr17\|56986968\|56987127\|+\|B2_Mm2\|Distal.upstream | 5.395589853 | 9.88E-05 |
| chr17\|44136883\|44137070\|+\|ID_B1\|Distal.downstream | 4.971086271 | 0.005197 |
| chr7\|103823699\|103823836\|-\|B1_Mus2\|Proximal.upstream | 4.882000548 | 0.001147 |
| chr17\|56987253\|56987342\|+\|PB1D7\|Proximal.upstream | 4.70013975 | 0.001548 |
| chr17\|56986737\|56986776\|+\|B2_Mm2\|Distal.upstream | 4.373860826 | 0.007666 |
| chr7\|63925318\|63925339\|+\|AT_rich\|intron | 4.232002059 | 0.000898 |
| chr17\|56987339\|56987519\|+\|B3A\|Proximal.upstream | 3.969093573 | 0.000313 |
| chr17\|56987571\|56987708\|-\|B1_Mur2\|Distal.upstream | 3.915010521 | 0.000236 |
| chrX\|106187084\|106187140\|+\|G-rich\|Proximal.upstream | 3.783022946 | 0.009826 |
| chr17\|56986517\|56986628\|+\|B1F\|Distal.upstream | 3.032369884 | 0.009477 |
| chr5\|146300326\|146300363\|+\|MIRc\|intron | 2.835385152 | 0.007805 |
| chr17\|56987961\|56988000\|-\|B1_Mus2\|Distal.upstream | 2.760463051 | 0.00192 |
| chrX\|68762820\|68762997\|-\|RSINE1\|intron | 2.596350207 | 0.000603 |
| chr10\|21311131\|21311158\|+\|AT_rich\|Proximal.downstream | 2.551765813 | 0.000427 |
| chr7\|63933068\|63933110\|+\|ORR1A2\|intron | 2.423580426 | 0.00671 |
| chr12\|99331328\|99331348\|+\|AT_rich\|intron | 2.412925315 | 0.00495 |
| chrX\|68803017\|68803167\|+\|MIRb\|intron | 2.231621674 | 0.003028 |
| chr17\|56986164\|56986250\|-\|PB1D7\|Distal.upstream | 2.202533246 | 0.004852 |
| chr14\|49020408\|49020429\|+\|AT_rich\|intron | 2.185343537 | 0.007695 |
| chr18\|35555903\|35555945\|+\|B1_Mur4\|intron | 2.06356627 | 0.006382 |
| chr17\|56989290\|56989434\|+\|B2_Mm1t\|Proximal.upstream | 1.980289497 | 0.003934 |
| chr8\|78140408\|78140529\|-\|ERVB4_1C-LTR_Mm\|Intergenic | 1.800719237 | 0.004946 |
| chr16\|45426639\|45426707\|+\|A-rich\|intron | 1.757629091 | 0.008767 |
| chr10\|80980501\|80980852\|+\|IAPLTR1_Mm\|intron | 1.739651919 | 0.003047 |
| chr13\|75453563\|75453735\|+\|MMERVK10D3_I-int\|Intergenic | 1.715715527 | 0.000297 |
| chr8\|128747108\|128747229\|-\|ERVB4_1C-LTR_Mm\|Proximal | 1.675812162 | 0.001344 |
| chr10\|80976038\|80976389\|+\|IAPLTR1_Mm\|intron | 1.671379146 | 0.00107 |
| chr6\|55026104\|55026808\|-\|IAPEz-int\|Distal.upstream | 1.664173254 | 0.009053 |
| chr16\|91255043\|91255195\|+\|ERVB4_1C-LTR_Mm\|Proximal.upstream | 1.644913488 | 0.001179 |
| chr2\|144155683\|144155835\|+\|ERVB4_1C-LTR_Mm\|Distal.upstream | 1.638164598 | 0.000882 |
| chr16\|25522498\|25522619\|+\|ERVB4_1C-LTR_Mm\|Intergenic | 1.604578511 | 0.001588 |
| chr1\|184828211\|184828231\|+\|AT_rich\|intron | 1.552152227 | 0.006177 |
| chr13\|75453728\|75454085\|+\|MMERVK10D3_I-int\|Intergenic | 1.532679379 | 0.003267 |
| chr17\|32819171\|32819261\|-\|MT2B\|3’ UTR | 1.488179662 | 0.008237 |
| chr7\|64126562\|64127302\|+\|RLTR4_Mm\|Distal.upstream | 1.484657595 | 0.000325 |
| chr12\|101677913\|101678039\|+\|B3A\|intron | 1.461086737 | 0.008731 |
| chr18\|75384729\|75384752\|+\|GC_rich\|intron | 1.424225468 | 0.00324 |
| chr16\|78977032\|78977094\|+\|T-rich\|intron | 1.413856415 | 0.006119 |
| chr18\|44739909\|44740144\|+\|RLTR13G\|intron | 1.373097803 | 0.005541 |
| chr12\|69546806\|69547546\|+\|RLTR4_Mm\|Intergenic | 1.362191397 | 0.000224 |
| chr12\|76450245\|76450569\|+\|ORR1A2\|Distal.downstream | 1.336274428 | 0.005655 |

| | | |
|---|---|---|
| chr6\|91881758\|91881828\|-\|MER2B\|intron | 1.31403406 | 0.006322 |
| chr11\|101449546\|101449596\|+\|B4\|intron | 1.298383004 | 0.002344 |
| chr6\|73293410\|73294150\|-\|RLTR4_Mm\|Distal.downstream | 1.293458747 | 0.000566 |
| chr5\|25230699\|25231439\|+\|RLTR4_Mm\|intron | 1.269462657 | 0.001097 |
| chr13\|56089407\|56089444\|+\|A-rich\|intron | 1.221031107 | 0.009064 |
| chr17\|56957693\|56957836\|-\|B1_Mur4\|intron | 1.214269375 | 0.008173 |
| chr11\|6745312\|6745554\|-\|RLTR13G\|Distal.upstream | 1.17477009 | 0.002964 |
| chr1\|45734427\|45735732\|+\|IAPEz-int\|Distal.upstream | 1.143417239 | 0.001176 |
| chr8\|105258102\|105258195\|-\|B1_Mur4\|Proximal.downstream | 1.133250173 | 0.006154 |
| chr8\|123164716\|123165456\|+\|RLTR4_Mm\|Proximal.upstream | 1.118516315 | 0.001593 |
| chr17\|14099214\|14099298\|+\|MMETn-int\|Intergenic | 1.102621791 | 0.002961 |
| chr7\|64134862\|64135602\|+\|RLTR4_Mm\|Distal.upstream | 1.039196848 | 0.003194 |
| chr14\|47531912\|47531949\|+\|AT_rich\|Proximal.downstream | 1.037025265 | 0.008436 |
| chr17\|46855595\|46855643\|+\|MER5A\|Distal.upstream | 1.032637208 | 0.00987 |
| chr4\|141208873\|141209083\|-\|IAPEz-int\|Distal.upstream | 1.029764766 | 0.006911 |
| chr3\|144720329\|144720355\|+\|GC_rich\|Proximal.upstream | 1.027540382 | 0.006416 |
| chr17\|56278593\|56278706\|-\|ID_B1\|Proximal.downstream | 1.02259412 | 0.003495 |
| chr15\|78524805\|78524849\|+\|G-rich\|3’ UTR | -2.497644156 | 0.00576 |
| chr8\|93977410\|93977438\|+\|AT_rich\|intron | -2.301630682 | 0.008895 |
| chr10\|13965971\|13965994\|+\|AT_rich\|Proximal.upstream | -2.154319953 | 0.006396 |
| chr11\|115156384\|115156480\|-\|PB1D9\|intron | -1.851326636 | 0.000786 |
| chr3\|90248161\|90248203\|+\|G-rich\|Proximal.upstream | -1.433803953 | 0.00625 |
| chr7\|100501389\|100501422\|+\|MT2B2\|3’ UTR | -1.431853033 | 0.007986 |
| chr4\|141619268\|141619356\|+\|CT-rich\|Proximal.upstream | -1.296053176 | 0.003 |
| chr7\|80241065\|80241113\|+\|B1F\|3’ UTR | -1.211871916 | 0.000569 |
| chr7\|120932828\|120932972\|-\|B1_Mus2\|intron | -1.097783417 | 0.002961 |
| chr15\|53209829\|53209966\|-\|Lx8b\|intron | -1.028704375 | 0.009269 |
| chr1\|192149183\|192149344\|-\|B3A\|non-coding | -1.025329162 | 0.005165 |
| chr1\|54477997\|54478109\|+\|B3A\|3’ UTR | -1.011851311 | 0.003043 |
