# Supplementary material for: Moderate DNA hypomethylation suppresses intestinal tumorigenesis by promoting caspase-3 expression and apoptosis
Source: Oncogenesis. 2021 May 4;10(5):38. doi: 10.1038/s41389-021-00328-9 (PMC8096944; doi:10.1038/s41389-021-00328-9)
Supplement: Supplementary file 4 — Supplementary Table S3 [file 41389_2021_328_MOESM4_ESM.pdf]

Supplementary Table S3. Primers for genotyping, qRT-PCR and bisulfite sequencing

**Primers for genotyping**	**Forward (5′-3′)**	**Reverse (5′-3′)**
Uhrf1$^{ki/ki}$ for WT	5'- TTTGCTGTCAGTAAACCTCTATG-3'	5'- GTCCACATACTCATTGACCTACG-3'
Uhrf1$^{ki/ki}$ for mutant	5'-GTGGCAGTATAGATTCTCACC-3'	5'-TATCGCCTTCTTGACGAGTTC-3'
Uhrf1$^{ki/ki}$ sequencing	5'- AGTTGCTGTCTCCCTTGTGC-3'	5'- GCTGAGCCTGGGCTTCTTCAC-3'
Apc for WT	5’-GCCATCCCTTCACGTTAG-3’	5’-TTCCACTTTGGCATAAGGC-3’(common)
Apc for *Apc*$^{min/+}$	5’-TTCTGAGAAAGACAGAAGTTA-3’	5’-TTCCACTTTGGCATAAGGC-3’ (common)
**Primers for qRT-PCR**	**Forward (5′-3′)**	**Reverse (5′-3′)**
Acer1	TCTGAGGTGGATTGGTGTGAG	TGAGGGGTCCAAAGATGAGGA
Ceacam10	TGCCACTACTGCCCAAGTC	GCCCCACTGTTTCCCTTGT
Fmrlmb	TGGTGGTGGCATGTAATAACC	CTTACTGGGGCATAGCATTTGAT
Mrs1	TTCACTGGATGCAATCTCCAAG	CTGGACTTCTGCTGATACTTTGT
Dpcr1	GAACGGGTCAGATGGAAAACG	GCTCTCATACCTGATTTCGTCGT
Slc4a11	ATGATGTCCTCCGAACTATGCT	TGTAAAGAGCTTAGCCATGAGC
Gapdh	ACCCAGAAGACTGTGGATGG	TTCAGCTCAGGGATGACCTT
Actin	GAGACCTTCAACACCCCAGC	ATGTCACGCACGATTTCCC
Caspase3	TGGTGATGAAGGGGTCATTTATG	TTCGGCTTTCCAGTCAGACTC
Caspase8	TGCTTGGACTACATCCCACAC	TGCAGTCTAGGAAGTTGACCA
Irf3	GAGAGCCGAACGAGGTTCAG	CTTCCAGGTTGACACGTCCG
Irf7	GAGACTGGCTATTGGGGGAG	GACCGAAATGCTTCCAGGG
Irf9	GCCGAGTGGTGGGTAAGAC	GCAAAGGCGCTGAACAAAGAG
Ifnb1	CAGCTCCAAGAAAGGACGAAC	GGCAGTGTAACTCTTCTGCAT
Stat1	TCACAGTGGTTCGAGCTTCAG	GCAAACGAGACATCATAGGCA
Isg15	GGTGTCCGTGACTAACTCCAT	TGGAAAGGGTAAGACCGTCCT
Lyz1	GAGACCGAAGCACCGACTATG	CGGTTTTGACATTGTGTTCGC
Lgr5	CCTACTCGAAGACTTACCCAGT	GCATTGGGGTGAATGATAGCA
Ascl2	AAGCACACCTTGACTGGTACG	AAGTGGACGTTTGCACCTTCA
Puma	TGTGGAGGAGGAGGAGTGG	TGCTGCTCTTCTTGTCTCCG

Fas	TTCTACTGCGATTCTCCTGGCTG	ATAGGCGATTTCTGGGACTTTGT
Fasl	CCGTGAGTTCACCAACCAAA	GGGGGTTCCCTGTTAAATGGG
Fadd	CTGCGCCGACACGATCTAC	CGGGCCAGTCTTTTCCAGT
Bax	GGACAGCAATATGGAGCTGCAGAGG	GGAGGAAGTCCAGTGTCCAGCC
**Primers for bisulfite sequencing**	**Forward (5′-3′)**	**Reverse (5′-3′)**
Enhancer-P1	TGGATAACCATGAAAACCTGAG	GAGGGATTGAGGAAGATACCCACC
Enhancer-P2	GTGCTGGGGATGCAAGCTCAGG	CCTCTAGAGCTAGAGTTACAAGTGGCAT
Enhancer-P3	TCTCCTGCTAGGAAAGGGAG	GGTGATTACCCTGCAAGCTTTACAGGGA
Enhancer-P4	CTAGGCATCACGGAAGCTTGG	GCTGTGCCATAAATCTAGGAAGGGG
Enhancer-P5	CGAGCCTTGCCTGGGGCAAG	TTTGTACTTGTTTGTACACAAAATTGGATC
Enhancer-P6	CCTGTCCCAGGAAGCAAGGGGG	TGGGTGGGTCCATCCCTGTTCC
Enhancer-P7	GACAGCCAGGGCTATACAGAGAAAGC	GGAACAGGGATGGACCCACCCAG
Promoter-P8	GGAGGTAATATAAAGGATTTTT	AAACCCTAAACTTACCCTAAATAAA
Promoter-P9	TTAGGTAAATATTGAAGGTAGAGAAGT	CAAATTCACCCCCACACTATAAC
